# Better Together Than Apart: Degradation of Emerging Contaminants in Concentrated Streams Using UV/Sulfite/Iodide and UV/Hydrogen Peroxide Treatment Trains

**DOI:** 10.1021/acsenvironau.6c00098

**Published:** 2026-07-01

**Authors:** Iván Sciscenko, Agustín París-Reche, Ana Agüera, Patricia Plaza-Bolaños, Claudio Minero, Marco Minella

**Affiliations:** † Department of Chemistry, University of Turin, Via Pietro Giuria 7, Turin 10125, Italy; ‡ Department of Chemistry and Physics, 16721 University of Almeria, Carretera de Sacramento s/n, Almería 04120, Spain; § Solar Energy Research Centre (CIESOL), Joint Centre of the University of Almeria-CIEMAT, Carretera de Sacramento s/n, Almería 04120, Spain

**Keywords:** advanced water treatment, hydrated electron, hydroxyl radical, per- and poly-fluoroalkyl substances (PFASs), photochemistry, retentate wastewater

## Abstract

This study evaluates the performance of homogeneous UV-based advanced reduction processes (ARPs) and advanced oxidation processes (AOPs), applied individually and in sequential treatment trains as a largely unexplored approach for the degradation of aqueous contaminants. UVC/SO_3_^2–^ and UVC/SO_3_^2–^/I^–^ were the reference ARPs, while UVC/H_2_O_2_ was used as the reference AOP. A mixture of three model contaminants was used in all cases, consisting of perfluorooctanoic acid (PFOA, as a model perfluoroalkyl substance—PFAS), ibuprofen, and benzoic acid. The UVC/SO_3_^2–^ treatment achieved 70% defluorination of 100 μM PFOA after 24 h of irradiation, while nearly complete defluorination was obtained with UVC/SO_3_^2–^/I^–^ in the same period. Benzoic acid exhibited the fastest degradation rates with both ARPs (*k*_obs_ = 3.9 × 10^–1^ min^–1^, 100 μM concentration), whereas ibuprofen was considerably slower, being comparable to its direct photolysis (*k*_obs_ = 2.9 × 10^–2^ min^–1^, 100 μM concentration). In contrast, the UVC/H_2_O_2_ process efficiently mineralized benzoic acid and ibuprofen (>99% within 2 h) but showed negligible degradation of PFOA (<1% in 2 h). The treatment trains (AOP → ARP and ARP → AOP) were decisively more efficient when dealing with the pollutant mixture degradation in the presence of scavengers. With the AOP → ARP approach, for matrices containing 50 mg L^–1^ of humic acids, the UVC/H_2_O_2_ pretreatment led to faster PFOA defluorination rates than ARPs alone (65% and 5% in 2 h, respectively). The inverse configuration, ARP → AOP, allowed the mineralization of PFOA, a result that could not be achieved when either ARPs or the AOP were applied individually. Experiments in simulated retentate wastewater, containing 1 μM of each contaminant and high concentration of anions and organic matter, were consistent with the preliminary results. This work is one of the first to offer valuable information on the degradation of PFAS and non-PFAS pollutants by the aforementioned treatment trains, especially in concentrated wastewater treatment streams.

## Graphical abstract

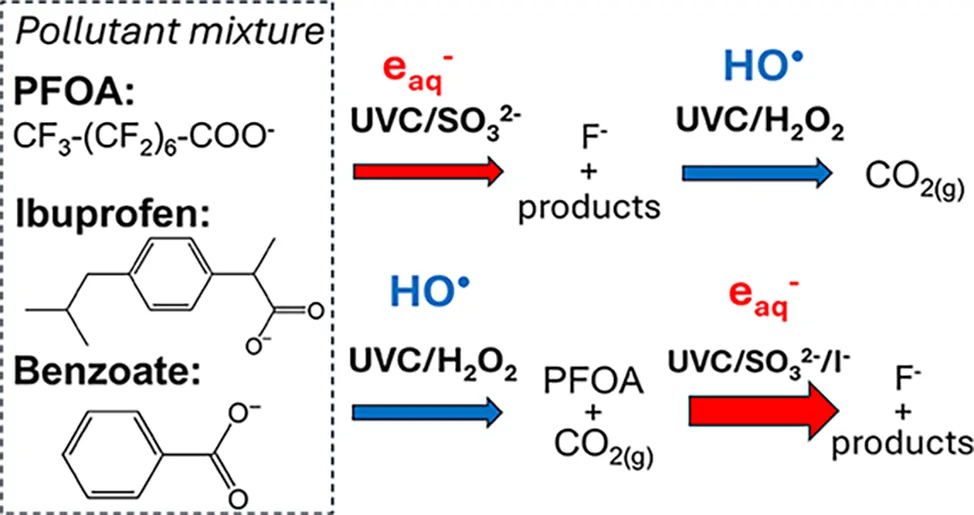

## 1 Introduction

Per- and poly-fluoroalkyl substances (PFASs) comprise a large and diverse group of synthetic compounds that are extensively produced by the chemical industry due to their use in numerous applications, including pesticides, fire extinguishing foams, lithium-ion batteries, solar panels, nonstick cookware, refrigerants, and textiles.^1^ One of the reasons for their remarkable applicability is that PFASs have extremely high thermodynamic stability, provided by the strong carbon–fluorine bonds (440–544 kJ mol^–1^).^2–4^ Along with their high persistence, the large-scale global production of PFASs, coupled with limited regulatory control and high mobility, has led to their widespread environmental occurrence,^5^ with indoor air (and dust), food, and drinking water being the main sources of PFASs for human intake.^6–8^ This is a serious concern regarding public health, because these substances have been associated with carcinogenicity, bioaccumulation, and alteration of reproductive and cognitive systems.^1,9^

To date, the most effective solution to remove PFASs from large water volumes relies on separation methods such as reverse osmosis, nanofiltration, granular/powdered activated carbon, anion exchange resins, and foam fractionation.^10,11^ However, the treatment of highly concentrated retentate streams (from filtration systems), PFAS-laden spent media (from adsorption systems), or exhausted foam remains an open challenge. Advanced reduction processes (ARPs), such as UVC/SO_3_^2–^ or UVC/I^–^, have gained momentum in the past few years as an efficient treatment to degrade PFASs up to fluoride ions and defluorinated byproducts.^12–15^ In ARPs, extremely reductive species, such as the hydrated electron (e_aq_^–^, *E*° = −2.9 V vs NHE), are generated.^16,17^ The reported bimolecular rate constants between e_aq_^–^ and these pollutants are in the range of 10^7^–10^9^ M^–1^ s^–1^,^18^ which is several orders of magnitude higher than those obtained with reactive species generated in traditional advanced oxidation processes (AOPs). For instance, the reported bimolecular rate constants of some PFASs with hydroxyl (HO^•^) or sulfate (SO_4_^•–^) radicals are <10^5^ M^–1^ s^–1^.^19,20^ The reason behind such a difference in reactivity is that HO^•^/SO_4_^•–^ have an electrophilic character,^21^ whereas the e_aq_^–^ has a nucleophilic one,^22^ making the positive electronic density of carbon atoms in PFASs an ideal target.

The first study in which ARPs were used for PFASs reduction was reported by H. Park et al.,^23^ employing the UVC/I^–^ process. They obtained fast PFASs degradations, with pseudo-first-order kinetic rate constants in the order of 1 × 10^–3^ min^–1^ (with a PFASs concentration between 20 and 30 μM). Moreover, they investigated the effect of chain length and the terminal groups (carboxylic and sulfonic). More recently, the group of J. Liu^12,24,25^ made important contributions to the fate of PFASs removal by e_aq_^–^ depending on its molecular structure as well as on optimizing the operational conditions. They also stated that, despite the fact that UVC/SO_3_^2–^ has a lower molar extinction coefficient and e_aq_^–^ photogeneration quantum yield than UVC/I^–^ (see reactions R1 and R2, respectively), the resulting e_aq_^–^ lifetime and, as a consequence, the PFASs abatement rates were significantly higher when employing the process with SO_3_^2–^ rather than I^–^.^26^ This is related to the fast e_aq_^–^ scavenging by the oxidation intermediates, I_2_, I_2_^•–^, and I_3_^–^ (see reactions R3–R5).^16^ Therefore, by combining SO_3_^2–^ and I^–^, the generated I_2_, I_2_^•–^, and I_3_^–^ react predominantly with SO_3_^2–^ (see reactions R6–R8), increasing the e_aq_^–^ lifetime and obtaining a synergy that allows faster PFASs degradation rates.^13^ Note that the formed ISO_3_^–^ in the UVC/SO_3_^2–^/I^–^ process is eventually hydrolyzed into I^–^ and SO_4_^2–^ (R9).

$$\begin{array}{c} {SO}_{3}^{2-}+h\nu \to {\text{SO}}_{3}^{•-}+e_{aq}^{-};\\ \Phi_{254nm}=0.15\;{\;\text{and}\;\;\varepsilon }_{254nm}=18\;M^{-1}\;{cm}^{-1} \end{array}$$ R1

$$\begin{array}{c} I^{-}+h\nu \to I^{•}+{e_{aq}}^{-};\\ \Phi_{254nm}=0.2\;{\;\text{and}\;\;\varepsilon }_{254nm}=220{\;M}^{-1}\;{cm}^{-1} \end{array}$$ R2

R3 $$I_{2}+{e_{aq}}^{-}\to {I_{2}}^{•-}$$

R4 $${I_{2}}^{•-}+{e_{aq}}^{-}\to 2I^{-}$$

R5 $${I_{3}}^{-}+{e_{aq}}^{-}\to I^{-}+{I_{2}}^{•-}$$

R6 $$I_{2}+{{\text{SO}}_{3}}^{2-}\to I^{-}+{{ISO}_{3}}^{-}$$

R7 $${I_{2}}^{•-}+{{\text{SO}}_{3}}^{2-}\to 2I^{-}+{{\text{SO}}_{3}}^{•-}$$

R8 $${I_{3}}^{-}+{{\text{SO}}_{3}}^{2-}\to 2I^{-}+{{ISO}_{3}}^{-}$$

R9 $${{ISO}_{3}}^{-}+H_{2}O\to I^{-}+{{\text{SO}}_{4}}^{2-}+2H^{+}$$

In spite of the good performances toward PFASs removal, restrictive operational conditions are required for UVC/SO_3_^2–^ and UVC/SO_3_^2–^/I^–^ treatments to be effective, such as high reagent concentrations, pH > 9, and absence of dissolved oxygen (i.e., anaerobic conditions). Therefore, ARPs are unlikely to be implemented at the same large scale as AOPs; however, they may be suitable for treating small volumes of concentrated contaminant waste streams.^16^ Since the concentrate-to-feed volume ratio typically ranges from 15 to 30% in nanofiltration systems and 15 to 60% in reverse osmosis ones, ARPs could represent a promising solution for the treatment of the generated concentrates.^27–29^ Noteworthily, to date, there are still few studies focusing on this topic.^10,30,31^ Furthermore, the transformation of PFASs into CO_2_ (mineralization) by ARPs is also very low, releasing defluorinated byproducts (e.g., carboxylic acids) into the effluent, which requires further assessment. In this sense, Zhang et al. recently employed UVC/SO_3_^2–^ as a pretreatment to defluorinate PFOA and mineralize the generated byproducts by a subsequent AOP, allowing nearly complete PFOA mineralization.^32^ However, the effect of interferences (such as NO_2_^–^ or HCO_3_^–^, strong HO^•^ scavengers)^33^ was not studied. Another interesting combination could be the inverse approach, i.e., an AOP pretreatment followed by an ARP post-treatment. Although this approach could lead to low PFASs mineralization percentages, it increases the e_aq_^–^ lifetime, since the AOP eliminates the dissolved organic matter (DOM) in the first step, which is considered a strong e_aq_^–^ scavenger.^30^

To date, there are no studies exploring the advantages of one approach over the other or situations in which it would be preferable to apply one or the other. This work intends to fill this gap by critically analyzing the performance of UVC/SO_3_^2–^ and UVC/SO_3_^2–^/I^–^ and their combination with a reference AOP, the UVC/H_2_O_2_.^34^ Since ARPs and the two possible treatment trains, AOP → ARP and ARP → AOP, should be suitable for application to concentrated streams, the effect of anions (NO_3_^–^, NO_2_^–^, HCO_3_^–^, HPO_4_^2–^, SO_4_^2–^, and Cl^–^), cations (Ca^2+^ and Mg^2+^), and DOM (humic acids and tryptophan) was investigated at high concentrations. For this purpose, a mixture of three model pollutants was employed: PFOA, ibuprofen (IB), and benzoic acid (BA). The first was used as a model PFASs, while the other two were used as non-PFASs emerging contaminants. IB is a pharmaceutical with a high occurrence in wastewater, and BA is a compound with natural and anthropogenic origins with also a high occurrence in wastewater effluents.^35–37^ Finally, the treatments investigated were studied to degrade the model contaminant mixture at trace levels in simulated retentate wastewater (SRWW).

## 2 Experimental Section

### 2.1 Reagents

The reagents employed in this work are listed in Table S1. Ultrapure water was obtained using a Merck-Millipore eq 7000 water system (total organic carbon –TOC– < 2 ppb, resistivity ≥18.2 MΩ cm). Standard solutions (H_2_O_2_ 1 M, BA 10 mM, IB 10 mM, and PFOA 1 mM) were stored at 4 °C; all of them were stable for at least a month. New Na_2_SO_3_ 1 M and KI 1 M stock solutions were prepared daily to prevent oxidation by dissolved oxygen.

### 2.2 Irradiation Experiments

Experiments were carried out in a similar way to that reported in previous works:^38^ 10 mL of sample was placed within closed cylindrical quartz cells (illuminated area 10 cm^2^), with a lateral neck for sample transfer and closed tightly with silicon septum caps. Each cell was placed under a UVC lamp (Hg low-pressure lamp Philips TUV T8 F17 1SL/25, 16.7 W). Irradiation was mainly from the top, and the distance between the cell and the lamp was 1 cm. The cells were taken out at different time intervals, and the agitation was provided by magnetic stirring. When anaerobic conditions were required, N_2_ was bubbled for 10 min prior to the irradiation through the septum cap of each reactor with a stainless-steel syringe (and an additional one for the gas exit). Each silicon septum was changed periodically to avoid air entrance. BA, IB, and PFOA were spiked at a concentration of 100 μM each in ultrapure water. No buffer solutions were employed in this work to avoid misinterpretations due to the anion effect, and pH was adjusted with NaOH or H_2_SO_4_ solutions. In all cases, standard deviations (when shown) were calculated from 2–4 replicates. The absence of standard deviation implies that no replicate was carried out for that specific sample.

#### 2.2.1 Advanced Reduction Processes

ARPs were performed by UVC/SO_3_^2–^, with and without I^–^, employing the conditions previously reported by Liu et al.:^26^ [SO_3_^2–^]_0_ = 10 mM, [I^–^]_0_ = 2 mM, and pH_0_ = 12.0. A design of experiment–response surface methodology at pH 12.0 varying SO_3_^2–^ and I^–^ concentrations confirmed that the optimal conditions were similar to the aforementioned (see Figure S1). The hindering effect of pH = 7 and dissolved oxygen was also confirmed (see Figure S2).

At pH 12, no significant pH changes were observed even after 24 h of irradiation (|ΔpH| < 0.2).

#### 2.2.2 Advanced Oxidation Process

Contrary to the ARPs, treatment with UVC/H_2_O_2_ was carried out at pH = 7.0 and in an aerobic atmosphere, employing a [H_2_O_2_]_0_ = 0.5 or 5 mM: the first is viable for large-scale water treatment applications,^39^ whereas the second is only applicable to concentrate streams.^40^ Experiments at pH 12 were avoided since they led to utterly lower pollutant abatement and mineralization rates (see Figure S3a), in line with H_2_O_2_ fast dismutation at those conditions, in spite of the higher molar extinction coefficient of HO_2_^–^ (p*K*_a_ = 11.6).^21^

The pH changes after 4 h of UVC/H_2_O_2_ treatment were slightly higher than those of ARPs due to the aerobic conditions employed (CO_2_ presence), the low buffer capacity due to H^+^/OH^–^ at the working pH, and the plausible formation of acidic byproducts. However, it was also nonsignificant (|ΔpH| < 0.5).

### 2.3 Effect of Water Constituents

As previously mentioned, since the retentates of membrane systems used for the removal of contaminants of emerging concern could have high anion and DOM concentrations, for both ARP and AOP, the effect of water constituents was studied by adding the desired species in low to high concentrations. Anions were studied within the 0.001–1 M range. The DOM content was simulated employing humic acid sodium salts and tryptophan within the 0.1–50 mg L^–1^ range. Due to the plausible formation of insoluble solids, the effects of Mg^2+^ and Ca^2+^ were also considered for the UVC/SO_3_^2–^/I^–^ process, studying their effects in the 1–10 mM range.

### 2.4 Treatment Trains

Since the UVC/H_2_O_2_ process was carried out at pH 7.0 and the UVC/SO_3_^2–^(/I^–^) at pH 12.0, a neutralization or a basification was required according to the studied configuration. When the pretreatment was the UVC/H_2_O_2_, SO_3_^2–^ and I^–^ were added to the final solution (which had a negligible H_2_O_2_ concentration, always confirmed before starting the subsequent step), the pH was then adjusted to 12.0, and anoxic conditions were achieved by N_2_ bubbling as previously described. Regarding the opposite treatment train, the ARP chosen was the one without I^–^ (see reasons in Section 3.4.1), which was neutralized with H_2_SO_4_ 0.5 M until pH 7.0, and the residual SO_3_^2–^ concentration was oxidized with a stoichiometric amount of H_2_O_2_. For further clarity, a scheme of the aforementioned procedure is summarized in Figure 1. Blank experiments containing 5 mM of SO_3_^2–^ and 10 mM of H_2_O_2_ were carried out to verify that no mineralization was obtained by the plausibly formed reactive species in the SO_3_^2–^/H_2_O_2_ system (e.g., SO_4_^•–^).^41^ It was also verified that the SO_3_^2–^/H_2_O_2_ reaction was unable to degrade BA, IB, or PFOA in a concentration of 1 or 100 μM.

**Figure 1:**
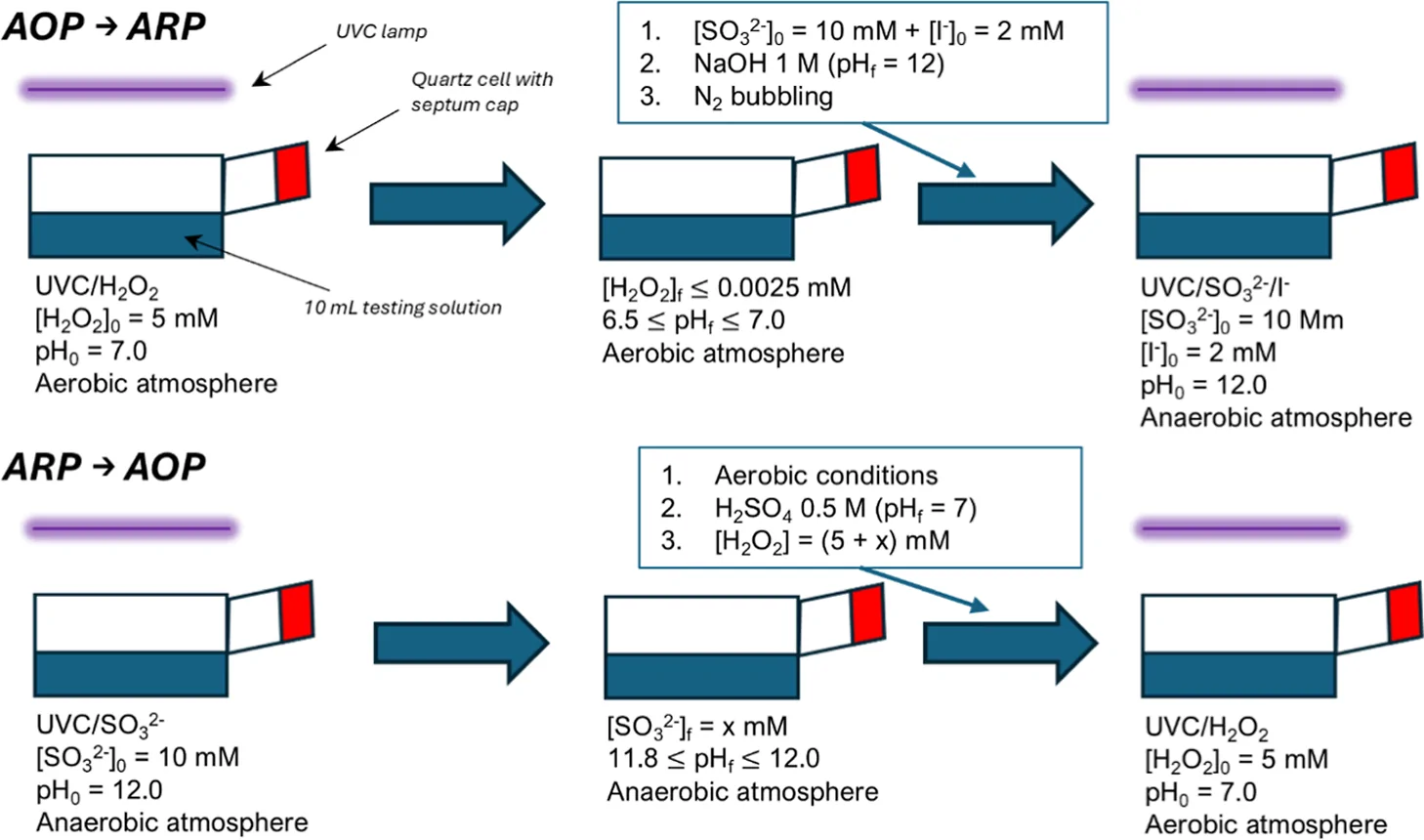
Methodological outline of the studied treatment trains.

### 2.5 Performance Evaluation in Simulated Retentate Wastewater

To gain further insights into the AOP and ARP treatments studied under more realistic conditions, their performance in degrading the aforementioned pollutant mixture at more realistic concentrations (1 μM each) was studied employing simulated retentate wastewater (SRWW), with its composition reported in Table 1. Ca^2+^ and Mg^2+^ salts’ use within the SRWW composition was avoided to circumvent the formation of insoluble precipitates; their effect is discussed in Section 3.3.2, where we demonstrated that they did not cause interferences in the studied processes.

**Table 1:** Simulated Retentate Wastewater (SRWW) Composition^a^

Reagent	Concentration
NaCl	35 mM
NaHCO_3_	10 mM
Na_2_SO_4_	5 mM
NaNO_3_	1.45 mM
NH_4_Cl	1.5 mM
NaNO_2_	0.6 mM
Na_2_HPO_4_•2H_2_O	0.1 mM
humic acid sodium salt	83 mg L^–1^ (27 ± 3 mgC L^–1^)
PFOA	0.001 mM
BA	0.001 mM
IB	0.001 mM

a The concentrations of the individual species were selected based on the values reported for retentates from reverse osmosis and nanofiltration systems employed to treat urban wastewater,^42^ paper mill wastewater,^43^ and surface and groundwater.^30,44^ The obtained SRWW exhibited a pH = 8.2 ± 0.1

### 2.6 Sample Management Prior to Analysis

For experiments carried out at pH 12.0, samples were neutralized prior to analysis to pH = 5–7 with H_2_SO_4_ 0.5 M. For the BA and IB analysis, the samples were previously passed through PTFE 0.20 μm syringe filters and collected in glass vials closed with PTFE septum caps (materials purchased from VWR). For the PFOA analysis, reverse cellulose 0.20 μm filters, polypropylene HPLC vials, and silicone HPLC-septum caps (purchased from Macherey Nagel) were used to avoid contamination of samples due to the ubiquity of this compound in common materials.

For the samples coming from the UVC/H_2_O_2_ treatment, a methanol excess was added (final concentration ca. 7% _V/V_) in order to quench any plausible pollutant oxidation by Fenton reaction due to transition metal trace contamination.^45^ For the mineralization measurements, an excess of Na_2_SO_3_ was employed to eliminate the residual H_2_O_2_ and stop all the potential oxidative reactions.

### 2.7 Analytical Measurements

For the 100 μM-range pollutant initial concentration experiments, BA, PFOA, and IB removals were measured by a HPLC-UV/vis (YL9300 HPLC) system, employing a C18 column (Lichrospher R100 5 μm) and a mobile phase of H_3_PO_4_ (5 mM) and acetonitrile in gradient mode: an 80:20 ratio (in % V/V) isocratic for the first 6 min, gradient until 60:40 in 4 min, isocratic 60:40 for 6 min, and return to the starting conditions in 2 min. The flow was always 1 mL min^–1^, and the employed wavelength was 200 nm, achieving the following retention times: 4.2 min for BA, 10.3 min for PFOA, and 15.2 min for IB.

IB and BA transformation products (TPs) were analyzed using an Agilent 1260 Infinity chromatographic system (Santa Clara, CA, USA) coupled to a QqTOF 5600+ hybrid mass spectrometer (AB Sciex, Foster City, CA, USA). Aliquots of 20 μL were injected onto a Kinetex PS C18 column (2.1 × 100 mm, 2.6 μm; Phenomenex, Torrance, CA, USA) maintained at 30 °C. The mobile phase consisted of water acidified with 0.1% formic acid (A) and methanol (B) delivered at 0.3 mL min^–1^. Gradient elution started at 90% A for 1 min, linearly increased to 100% B over 11 min (held for 4 min), returned to 90% A over 4 min, and re-equilibrated for 10 min (total run time: 30 min). Analyses were conducted in negative ESI mode with source parameters: spray voltage: 4500 V; GS1 and GS2: 60 psi; curtain gas: 30 psi; and source temperature: 575 °C. Data acquisition combined full-scan TOF-MS (*m*/*z* 100–1000, 0.25 s accumulation) and data-dependent acquisition (DDA) targeting the four most intense ions (MS/MS collision energy 35 V; dynamic exclusion 3 s). Mass calibration was performed every five injections (<2 ppm accuracy). Raw data were processed with PeakView software (v2.2; AB Sciex). PFOA TPs were analyzed using similar UHPLC-HRMS conditions, except that 10 μL was injected and mobile phase A contained 20 mM ammonium acetate instead of 0.1% formic acid, and the gradient elution profile was as follows: 90% A for 1 min, linearly increased to 100% B over 12 min (held for 8 min), returned to 90% A over 2 min, followed by 9 min re-equilibration (total run time: 31 min). A suspect screening strategy was applied for the identification of TPs. Three suspect lists were created including accurate mass information on TPs selected, based on a literature review for the compounds investigated. The requirements for peak extraction were as follows: intensity threshold >1000 cps and *S*/*N* > 10. Tentative identification was based on the following criteria: mass accuracy error <5 ppm for the molecular ion, isotopic ratio difference <10%, correct formula assignment by Formula Finder software, and MS/MS spectra concordance with databases (fit >70%) or literature.^46–48^ Final confirmation of positive findings was obtained by analytical standards when available.

For the 1 μM concentration experiments, the IB and BA abatement rates were measured using an UHPLC system (Exion LC AC, Sciex, Foster City, CA, USA) coupled to a Triple Quad7500 mass spectrometer (AB Sciex Instruments, Wilmington, DE, USA). The UHPLC was equipped with a Luna Omega Polar C18 10 LC analytical column (100 × 2.1 mm, 1.6 μm particle size, Phenomenex, Torrance, CA, USA) operated at a flow rate of 0.3 mL min^–1^. The mobile phases consisted of (A) LC–MS grade water containing 0.1% formic acid and (B) LC–MS grade methanol. The initial proportion of solvent B was set at 5% for 1 min, increased to 100% within 8 min, held constant for 4 min, and then decreased back to 5% in 0.5 min. The equilibration time was 5 min, resulting in a total run time of 17.5 min. The samples were directly injected into the LC–MS system. The MS used a Turbo IonSpray interface operated in negative mode. The ionization source parameters were as follows: curtain gas, 40 psi; collision gas (CAD), 9 psi; ion spray voltage, 2000 V; temperature, 350 °C; gas 1, 50 psi; and gas 2, 70 psi. The MS operated in multiple reaction monitoring (MRM) acquisition mode, and Sciex OS 3.1.6.44 software was used for data acquisition and data processing. On the other hand, the PFOA was analyzed on the same UHPLC–MS/MS system, using a Luna Omega PS C18 column (100 × 3.0 mm, 3 μm, Phenomenex), with a Gemini C18 retention column (50 × 4.6 mm, 3 μm) installed before the injector to minimize PFASs contamination. The mobile phases were (A) 20 mM ammonium acetate in water (A) and methanol (B), with a gradient: 10% B (1.5 min), linear increase to 99% B (8 min), maintenance for 2 min, return to initial conditions in 0.5 min, and maintenance for 2.5 min.

The TOC, inorganic carbon (IC), and total nitrogen (TN) measurements were carried out by means of a Shimadzu TOC-V CSH total organic carbon analyzer.

The concentration of anions was measured by a Dionex DX 500 Ion Chromatograph equipped with an ED-40 conductometric detector, a Dionex IonPac AS9-HC column (4 × 250 mm), and a Thermo-Scientific ADRS 600 4 mm conductivity suppression unit, employing 0.8 mL min^–1^ of K_2_CO_3_ 9 mM mobile phase. Defluorination was calculated based on the measured F^–^ concentration at a certain time and the total stoichiometric fluoride contained in the initial PFOA concentration (1 mol of PFOA equivalent to 15 mol of fluoride).

The H_2_O_2_ concentration was measured spectrophotometrically by using a colorimetric method previously reported,^38^ employing phenol, peroxidase, phosphate buffer pH 7.2, and 4-aminoantipyrine as reagents, measuring the resulting absorbance at 505 nm.

### 2.8 Actinometric Measurements

The UVC irradiance and photon flux were measured periodically according to the well-known I^–^/IO_3_^–^ actinometric method described in the work by Rahn et al.:^49^ each quartz cell was filled with 10 mL of a solution containing [I^–^] = 0.60 M, [IO_3_^–^] = 0.10 M, and [borax] = 0.010 M and irradiated for different periods from 1 to 10 min, measuring the concentration of the formed I_3_^–^ at 352 nm with a Shimadzu UV-2600i UV–vis spectrophotometer (optical path, 1 cm, and I_3_^–^ molar extinction coefficient at 352 nm 27,600 L mol^–1^ cm^–1^).

The UVC lamp irradiance (*H*) was determined as 2.6 ± 0.2 mW cm^–2^, calculated from eq 1, where *E*_254 nm_ is the energy of a mol of photons at 254 nm (equivalent to 4.716 × 10^5^ J mol^–1^ or J Einstein^–1^), [I_3_^–^]_352 nm_ the corresponding I_3_^–^ concentration, *t* the irradiation time (s), Φ­(I_3_^–^)_352 nm_ the I_3_^–^ formation quantum yield (equivalent to 0.73 in the employed system), *V* the irradiated volume (0.01 L), and a the illuminated area (10 cm^2^).

On the other hand, the photon flux (*q* ^0^) was calculated according to eq 2,^50^ where ε_254 nm_ is the I^–^ molar extinction coefficient at 254 nm (172 L mol^–1^ cm^–1^), [I^–^]_0_ = 0.60 M, and *l* the optical path from the 10 mL solution within the quartz cell, which was 1 cm. In this way, the *q* ^0^ was determined to be 5.6 ± 0.4 × 10^–8^ Einstein s^–1^.

1 $$H=E_{254nm}\frac{\left[I_{3}^{-}\right]}{t}\frac{1}{\Phi {\left(I_{3}^{-}\right)}_{352nm}}\frac{V}{a}$$

2 $$q^{0}=\frac{\left[I_{3}^{-}\right]}{t}\frac{1}{\Phi {\left(I_{3}^{-}\right)}_{352nm}}\frac{V}{\left(1-10^{-\varepsilon_{254nm}{\left[I^{-}\right]}_{0}l}\right)}$$

### 2.9 Electrical Energy per Order Calculation

When needed, the electrical energy per order (*E*_EO_, expressed in kW h m^–3^), the electrical energy required to reduce the pollutant concentration by 1 order of magnitude per m^3^ of water, was calculated according to eq 3:

$$E_{EO}=\frac{Pt}{V}=1670\;kWm^{-3}t$$ 3

where *P* is the lamp’s electrical consumption (0.0167 kW), *t* the time, in hours, necessary to degrade a contaminant by 90%, and *V* the employed volume (1 × 10^–5^ m^3^). *E*_EO_ is a commonly used parameter to compare ARP or AOP performances.^12,51^

## 3 Results and Discussion

### 3.1 Advanced Reduction Processes: UVC/SO_3_^2–^ and UVC/SO_3_^2–^/I^–^

Figure 2a shows the degradation kinetics of the three pollutants by UVC, UVC/SO_3_^2–^, and UVC/SO_3_^2–^/I^–^. Regarding the PFOA (not degraded by UVC alone), the UVC/SO_3_^2–^/I^–^ treatment led to faster degradation than UVC/SO_3_^2–^: *k*_obs_ = 7.2 ± 0.1 × 10^–2^ min^–1^ and 4.8 ± 0.5 × 10^–2^ min^–1^, respectively, in line with the literature.^13,15,26,52^ As for the non-PFASs pollutants, IB and BA showed opposite behaviors. For IB, no significant differences were observed by UVC or UVC/SO_3_^2–^ (*k*_obs_ = 2.9 ± 0.1 × 10^–2^ min^–1^), while the I^–^ addition to the latter only enhanced the removal rate by a small extent (*k*_obs_ = 4.9 ± 0.1 × 10^–2^ min^–1^). On the contrary, for BA (whose photolysis was negligible, *k*_obs_ = 6.0 ± 0.7 × 10^–3^ min^–1^), the UVC/SO_3_^2–^ and UVC/SO_3_^2–^/I^–^ led to outstandingly fast removals, observing a *k*_obs_ = 3.9 ± 0.5 × 10^–1^ min^–1^ in both cases, the contribution of I^–^ not being noticeable.

**Figure 2:**
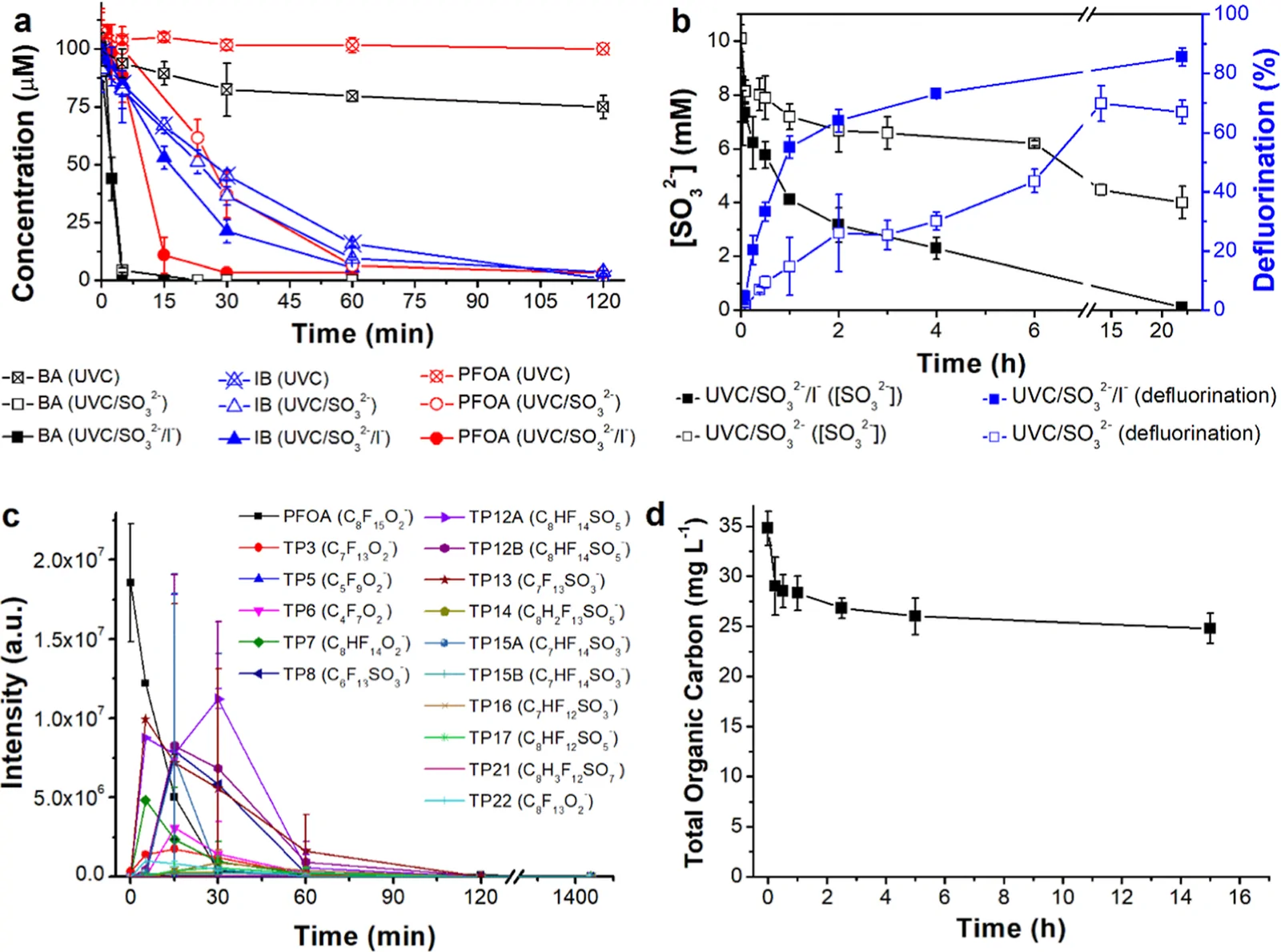
Results from the studied ARPs, UVC/SO_3_^2–^/I^–^, and UVC/SO_3_^2–^: (a) pollutant degradation kinetics (also including those by UVC alone); (b) SO_3_^2–^ concentration (*Y*-left axis, results in black) and PFOA defluorination percentage (*Y*-right axis, results in blue) over time; (c) PFOA major degradation byproduct profiles during the UVC/SO_3_^2–^/I^–^ process; (d) total organic carbon decay over time during the UVC/SO_3_^2–^/I^–^ process (TOC contribution from each pollutant: BA, 8.4 mg L^–1^; IB, 15.6 mg L^–1^; and PFOA, 9.6 mg L^–1^). Conditions: ultrapure water adjusted at pH 12.0, N_2_ atmosphere, [SO_3_^2–^]_0_ = 10 mM, and [I^–^]_0_ = 2 mM. In all cases, the starting solution contained 100 μM of BA, IB, and PFOA, respectively.

The observed differences between IB and BA degradation rates are in line with the fact that the reactivity of aromatic compounds with e_aq_^–^ is determined by the same parameters that control the nucleophilic aromatic substitution rates, being enhanced by electron-withdrawing groups (−COO^–^ for BA) and hindered by electron-donating ones (−CH_2_CH_2_(CH_3_)_2_ and –CH­(CH_3_)­COO^–^ for IB).^22^ Therefore, it is reasonable that the kinetics obtained are consistent with the reported bimolecular rate constants with e_aq_^–^: BA (3 × 10^9^ M^–1^ s^–1^) > PFOA (7 × 10^8^ M^–1^ s^–1^) > IB (estimated to be ≤2 × 10^7^ M^–1^ s^–1^ based on similar compounds).^53,54^

The catalytic effect of I^–^ on the UVC/SO_3_^2–^ process was also noticeable when analyzing the F^–^ and SO_3_^2–^ concentration trends (see Figure 2b). After 4 h of treatment, while the UVC/SO_3_^2–^ led to a 30 ± 3% defluorination, the UVC/SO_3_^2–^/I^–^ produced a 73 ± 1%, obtaining an overall defluorination of 85 ± 3% in 24 h.

The SO_3_^2–^ consumption was also faster when adding I^–^ (55% in 24 h when employing UVC/SO_3_^2–^ and >99% in the same period for UVC/SO_3_^2–^/I^–^). In fact, the initial SO_3_^2–^ concentration was lower when employing the UVC/SO_3_^2–^/I^–^ process: 10.1 ± 0.5 mM when applying UVC/SO_3_^2–^ and 8.5 ± 0.4 mM with the latter. These observations are consistent with the mechanism described in R6–R9 and with the fact that I^–^ accelerates the oxidation of SO_3_^2–^ by O_2_.^55^ As expected, the I^–^ concentration remained practically constant during the whole duration of the experiment (<10% oxidation, some I_3_^–^ detected after 24 h of irradiation in line with the consumption of SO_3_^2–^).

The degradation of PFOA followed the widely reported stepwise reaction due to e_aq_^–^.^12,23,24,56–59^ Up to 17 transformation products (TPs) could be tentatively identified, and 5 of them were confirmed with the analytical standard. Table S2 shows the list of TPs investigated and reported in literature, highlighting in bold those identified in this study. They corresponded with the progressive formation of shorter-chain perfluoro-carboxylic acids (perfluoroheptanoic acid (TP3), perfluoropentanoic acid (TP5), and perfluorobutanoic acid (TP6)); the formation of perfluoro-sulfonates (perfluorohexanesulfonic acid (TP8) and perfluoropentanesulfonic acid (TP20)); and the defluorination mechanisms by H–F exchange (TP7, TP10, TP13, TP23, TP15, TP16, and TP17) and ^•^SO_3_–F exchange (TP12A, TP12B, TP14, and TP17) in both perfluoro-carboxylic and perfluoro-sulfonate derivatives. As shown in Figure 2c, most of these transformation products were degraded within the first 2 h, which explains the observed change in the defluorination rate right afterwards. Regarding the TPs of BA and IB, no stable compounds were observed at the HRMS. It is important to highlight that no iodinated byproducts were detected, in line with other works.^26,60^

Although the better performance of UVC/SO_3_^2–^/I^–^ than UVC/SO_3_^2–^ has been widely reported, most of these works stated near-quantitative defluorinations of PFOA (>95%),^24,25^ whereas in our study, it remained 15% below. This might be explained by the formation of gaseous PFASs due to the attack of the e_aq_^–^ on the carboxylic acid moiety of PFOA and/or its generated byproducts, as demonstrated in past studies.^23,32^ In fact, 17% TOC reduction was obtained at the first 15 min of the UVC/SO_3_^2–^/I^–^ process, which continued diminishing with a less marked rate, reaching 30% after 15 h of irradiation (see Figure 2d). Although conversion of the studied pollutants into CO_2_ should not be disregarded, a similar issue might happen to BA or IB, generating gaseous products which diminish the TOC over time. Another possibility that cannot be excluded to explain the incomplete PFOA defluorination is that some of the formed PFASs are more recalcitrant to defluorination (e.g., TP8 or TP16), as reported in other works.^24^

### 3.2 Advanced Oxidation Process: UVC/H_2_O_2_

The behavior of the pollutant mixture under the chosen AOP, UVC/H_2_O_2_, was studied employing low (0.5 mM) and high (5 mM) peroxide concentrations. Although the treatment was able to degrade >99% of BA and IB in 30 min when employing 0.5 mM of H_2_O_2_ (*k*_obs_ = 2 × 10^–1^ min^–1^ in both cases), the PFOA oxidation was negligible for at least 1 h of irradiation (see Figure S3b). Degradations were, therefore, in line with reported bimolecular rate constants of the model pollutants with HO^•^, being 1 × 10^10^ M^–1^ s^–1^ for IB, 6 × 10^9^ M^–1^ s^–1^ for BA, and <10^5^ M^–1^ s^–1^ for PFOA.^19,53,61^ The blank control (pollutant photolysis kinetics at pH 7 and aerobic conditions) is shown in Figure S4, the results being in line with the vast literature on the matter:^61–63^ BA with 80% degradation in 1 h and IB with 95% degradation in 1 h, with the PFOA concentration remaining constant.

Regarding the mineralization, a plateau of 60% was reached in 4 h, corresponding to the TOC decay from BA and IB, with the remaining 40% corresponding to PFOA (see Figure S3c). The PFOA remained undegraded even with a 10 times higher H_2_O_2_ concentration (Figure 3a). With these conditions, the BA and IB were mineralized in 1 h, with no improvements for PFOA after a second addition of H_2_O_2_ 5 mM after 2 h of treatment (Figure 3b). Because of the faster mineralization kinetics with 5 mM of H_2_O_2_, this concentration was the one employed hereafter for the UVC/H_2_O_2_ process.

**Figure 3:**
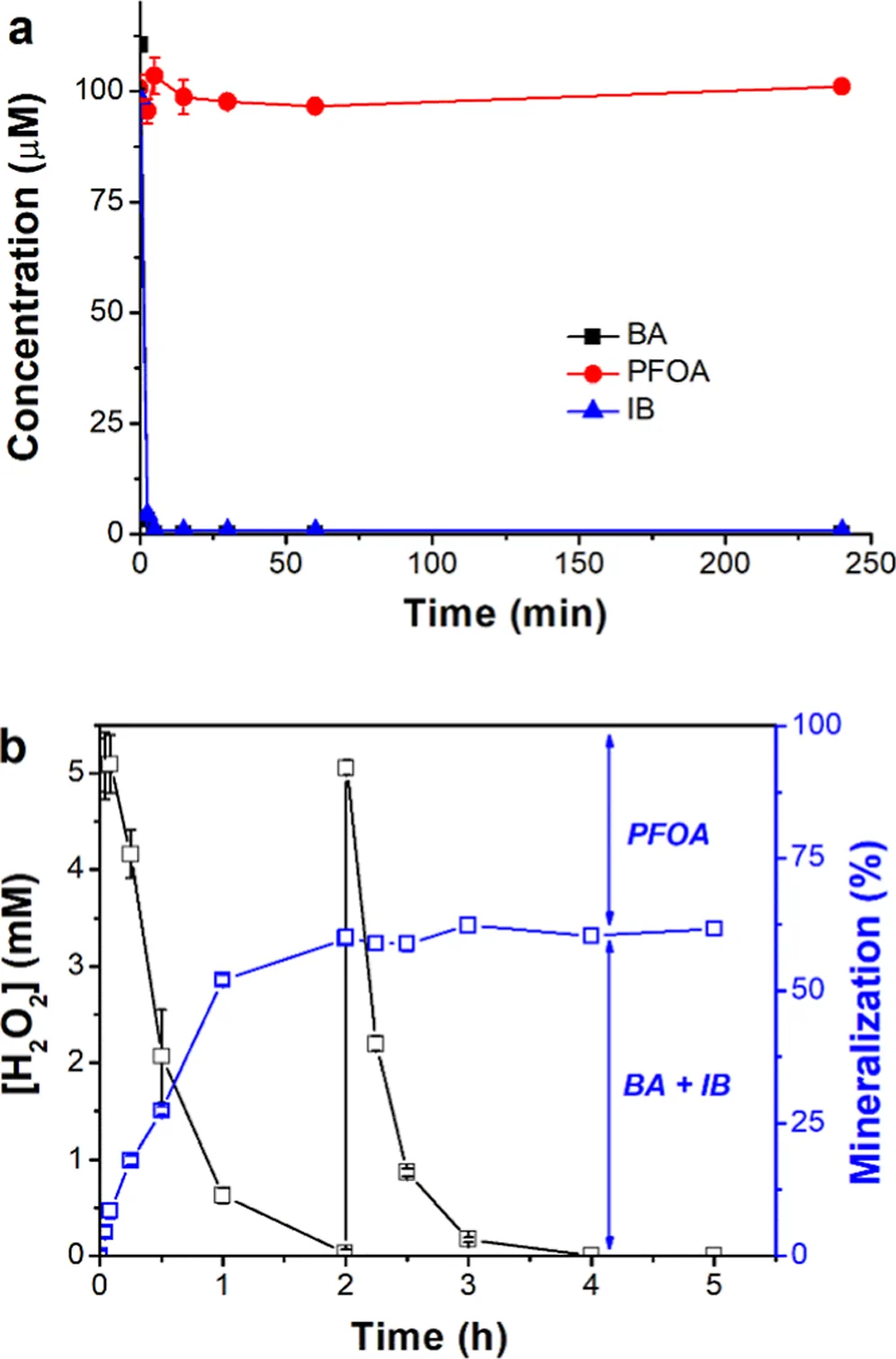
Results from the UVC/H_2_O_2_ process: (a) pollutant oxidation kinetics and (b) H_2_O_2_ consumption (*Y*-axis on the left, results exhibited in black) and mineralization (*Y*-axis on the right, results exhibited in blue). Conditions: ultrapure water adjusted at pH_0_ = 7.0, aerobic atmosphere, [H_2_O_2_]_0_ = 5 mM, with a second addition after 2 h. In all cases, the starting solution contained 100 μM of BA, IB, and PFOA, respectively.

### 3.3 Effect of Water Matrices on UVC/SO_3_^2–^/I^–^ and UVC/H_2_O_2_ Processes

#### 3.3.1 Anions

The effect of anions was first evaluated at 1 mM concentration each. As shown in Figures S5a and S6a, NO_2_^–^ produced the highest hindering effect to both oxidative and reductive treatments, leading to a mineralization of barely 10% in 1 h with the UVC/H_2_O_2_ process and 5% defluorination in 30 min by UVC/SO_3_^2–^/I^–^. This is in line with the intermediate redox state of NO_2_^–^: easily oxidizable and reducible, reflected in its high rate constants with HO^•^ and e_aq_^–^ (1 × 10^10^ M^–1^ s^–1^ and 4 × 10^9^ M^–1^ s^–1^, respectively).^53^ Indeed, if we estimate the HO^•^ and e_aq_^–^ steady-state concentrations in the absence and in the presence of NO_2_^–^ 1 mM, we find that in the AOP, the steady-state concentration of HO^•^ is 7.25 times higher in the absence of NO_2_^–^, whereas for the ARP, the steady-state concentration of e_aq_^–^ in the absence of NO_2_^–^ is 11.75 times higher (see the deduction in Text S1).

Regarding the other anions, the inorganic carbon mostly affected the AOP’s performance, in line with the fact that HCO_3_^–^ is a typical HO^•^ quencher (8.5 × 10^6^ M^–1^ s^–1^), whereas CO_3_^2–^ has low reactivity with e_aq_^–^ (1 × 10^5^ M^–1^ s^–1^).^64,65^ Opposite to carbonates, NO_3_^–^ only affected the ARP, as it is a well-known e_aq_^–^ scavenger (1 × 10^10^ M^–1^ s^–1^), being inert against HO^•^.^66^ Phosphates, Cl^–^, and SO_4_^2–^ did not affect the studied processes significantly, in agreement with their scarce reactivity toward the produced reactive species.^64,66^ In addition, since Cl^–^ is usually the anion with the highest concentrations in retentate wastewater (see Table 1), its effect until 1 M concentration was investigated. However, as shown in Figures S5b and S6b, no significant scavenging was observed for either UVC/SO_3_^2–^/I^–^ or UVC/H_2_O_2_.

Attention was placed on the fate of NO_2_^–^ and NO_3_^–^ due to the significant scavenging effect produced in the studied processes and because they are well-known contaminants with high occurrence in different water bodies.^67,68^ In this sense, the ARPs can eliminate both, whereas the AOPs can oxidize NO_2_^–^ into (among others) NO_3_^–^,^69^ hence, the ARP is more convenient from an environmental point of view.

The NO_3_^–^ and NO_2_^–^ 1 mM removal by UVC/SO_3_^2–^/I^–^ is shown in Figures 4a and 4b, respectively (results from blank experiments in the absence of SO_3_^2–^ and I^–^ are shown in Figure S7). Regarding the NO_3_^–^ 1 mM, its disappearance was correlated with the concomitant generation of NO_2_^–^, observing its maximum after 5 min of treatment, shortly after being reduced; the NO_3_^–^ and NO_2_^–^ concentrations were negligible in less than 1 h of treatment. For the case of NO_2_^–^ 1 mM, its abatement was >99% in 30 min. In agreement with the NO_2_^–^/NO_3_^–^ removals, the pollutant abatement for PFOA and IB was slow for the first 30 min (when the NO_2_^–^/NO_3_^–^ concentration was considerable) and fast for the next 30 min (see Figures 4c and 4d), in line with other works.^30^ This is relevant in terms of treatment costs. For instance, for the PFOA abatement, while in the absence of anions the estimated *E*_EO_ was 420 kW h m^–3^, with NO_3_^–^ or NO_2_^–^ it increased to ca. 1670 kW h m^–3^, which is almost 4 times higher. For the BA, on the contrary, due to its higher reactivity toward e_aq_^–^, its removal rate was slightly diminished by the presence of the aforementioned.

**Figure 4:**
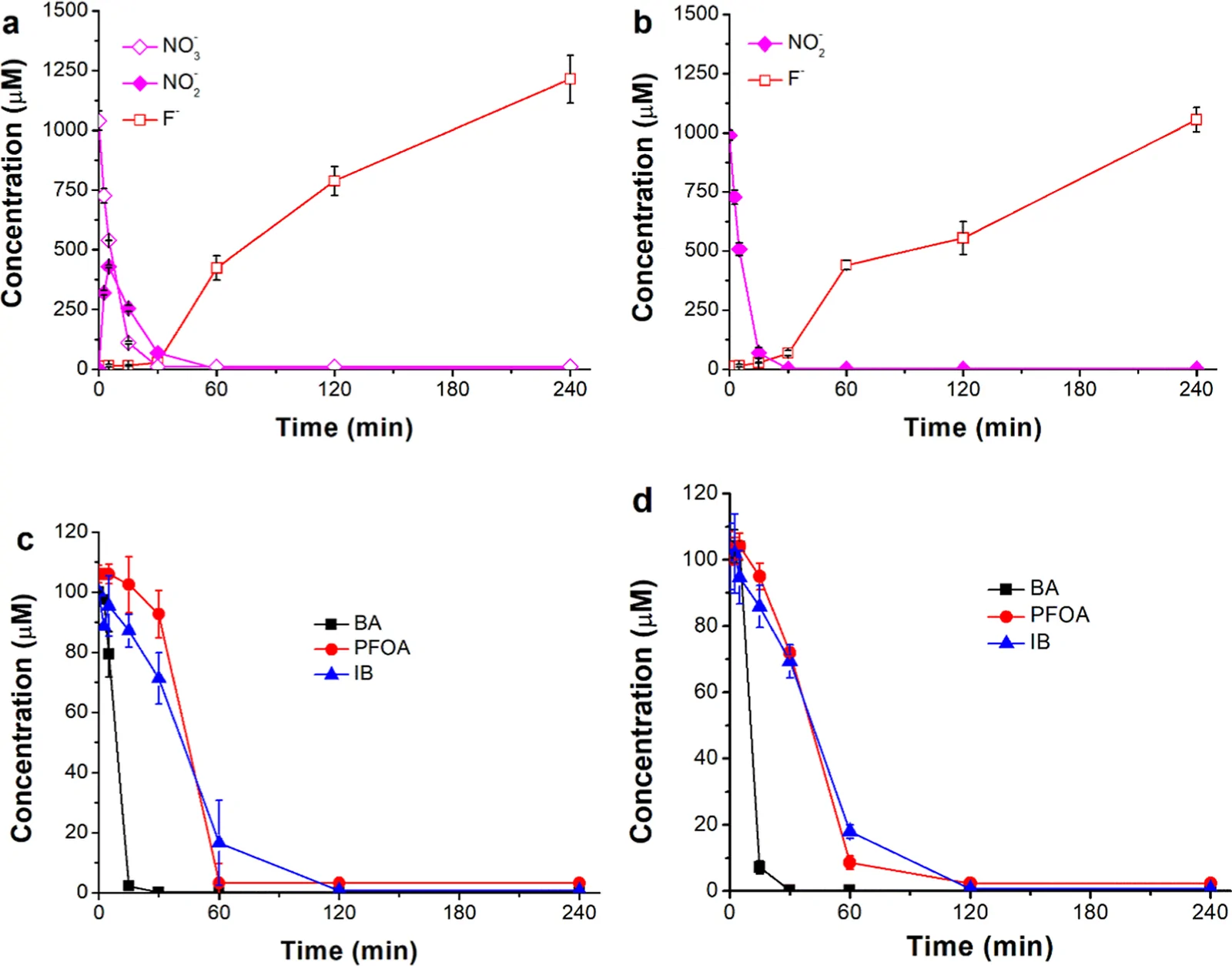
Effect of 1 mM of NO_3_^–^ or NO_2_^–^ in the UVC/SO_3_^2–^/I^–^ process: (a) ion concentration and (c) contaminant kinetics in the presence of NO_3_^–^ 1 mM; (b) ion concentration and (d) contaminant degradation kinetics in the presence of NO_2_^–^ 1 mM. Conditions: [SO_3_^2–^]_0_ = 10 mM, [I^–^]_0_ = 2 mM, pH_0_ = 12.0, N_2_ atmosphere. In all cases, the starting solution contained 100 μM of BA, IB, and PFOA, respectively.

To verify if NO_2_^–^ and NO_3_^–^ were transformed into gaseous products (e.g., N_2_O or N_2_), the TN was measured over time. As shown in Figure S8a, a fast TN decay was observed during the first 30 min of the experiment (25% with NO_3_^–^ and 50% with NO_2_^–^), remaining constant thereafter. Regarding results published in the literature, one might find opposite results: while some report the complete NO_2_^–^/NO_3_^–^ reduction until N_2_ by these ARPs,^70^ others state their reduction into N-intermediates rather than N_2_,^71^ the latter being more consistent with our results. Respecting the SO_3_^2–^ consumption, it did not differ from that in ultrapure water (see Figure S8b).

#### 3.3.2 Calcium and Magnesium

Filtration or reverse osmosis retentates are likely to contain Ca^2+^ and Mg^2+^ concentrations within the range of 1–10 mM.^30,43,44,72^ Although these cations are not expected to significantly interfere with the UVC/H_2_O_2_ process, they may affect the ARPs through precipitation of SO_3_^2–^ or F^–^ species. Since Cl^–^ did not affect the ARPs, CaCl_2_ and MgCl_2_ salts were used in each case.

Based on the obtained pollutant abatement kinetics and defluorination rates, neither Ca^2+^ nor Mg^2+^ affected the UVC/SO_3_^2–^/I^–^ performance up to 1 mM concentration (see Figure S9a). However, with 10 mM concentration, each cation produced different effects.

With Mg^2+^ 10 mM, even though the BA, IB, and PFOA removals were analogous to the ones obtained in its absence, the defluorination rate was slightly slower (26 ± 3% in 30 min, 1.4 times less than the one obtained in ultrapure water). This was due to the precipitation of MgF_2_ (K_sp_ = 8.5 × 10^–9^)^73^ that could limit the concentration of fluoride in the solution; by measuring the same samples with the addition of EDTA 20 mM previous to filtration, the F^–^ concentration was comparable to the ones obtained without Mg^2+^, indicating that it does not affect the actual PFOA defluorination rate but rather introduces an analytical interference that should be considered during fluoride quantification. It is also worth mentioning that, due to the precipitation of Mg­(OH)_2_ (*K*_sp_ = 1.5 × 10^–11^), higher NaOH amounts were required to reach the initial pH = 12 with respect to ultrapure water.

By contrast, Ca^2+^ 10 mM produced a fast precipitation of SO_3_^2–^ (CaSO_3_ *K*_sp_ = 7 × 10^–8^),^74^ evidenced by the formation of white crystals and a sharp decrease of SO_3_^2–^ concentration to 1.1 ± 0.3 mM in barely 30 min (see Figure S9b). No immediate CaSO_3_ precipitation was observed, as the starting SO_3_^2–^ concentration was similar to that measured in the absence of Ca^2+^. To verify if CaF_2_ (*K*_sp_ = 3 × 10^–11^) and CaSO_4_ (*K*_sp_ = 9 × 10^–6^) were formed,^73,74^ EDTA 20 mM was added into the solution, but the F^–^ and SO_4_^2–^ signals remained identical to the one without the ligand addition, only observing higher intensity for the SO_3_^2–^ signal as expected.

Interestingly, despite the SO_3_^2–^ concentration becoming negligible after 1 h of treatment in the system containing 10 mM of Ca^2+^, the PFOA defluorination rate remained comparable to the one obtained in ultrapure water, whereas the IB degradation was even faster (see Figure S9c); for the BA and PFOA kinetics, no relevant differences were observed. These results suggest that the UVC/CaSO_3_ system is also capable of generating e_aq_^–^. Some recent works also reported water treatment enhancements by using CaSO_3_ instead of Na_2_SO_3_,^74,75^ demonstrating that Ca^2+^ shall not suppose an interference to the ARPs and might even favor the pollutant degradation.

#### 3.3.3 Effect of Dissolved Organic Matter

Although the characteristics of DOM vary greatly among water sources, humic acids are a major component of natural organic matter, being commonly used to simulate their effects.^45^ The repercussion of increasing the humic acid concentration on the UVC/SO_3_^2–^/I^–^ process is shown in Figure 5a. An exponential hindering effect was observed by increasing its concentration from 0.1 mg L^–1^ to 25 mg L^–1^, the PFOA defluorination being negligible above 25 mg L^–1^ (see Figure S10a), and 50 mg L^–1^ of humic acids being sufficient to almost hinder BA removal in 30 min.

**Figure 5:**
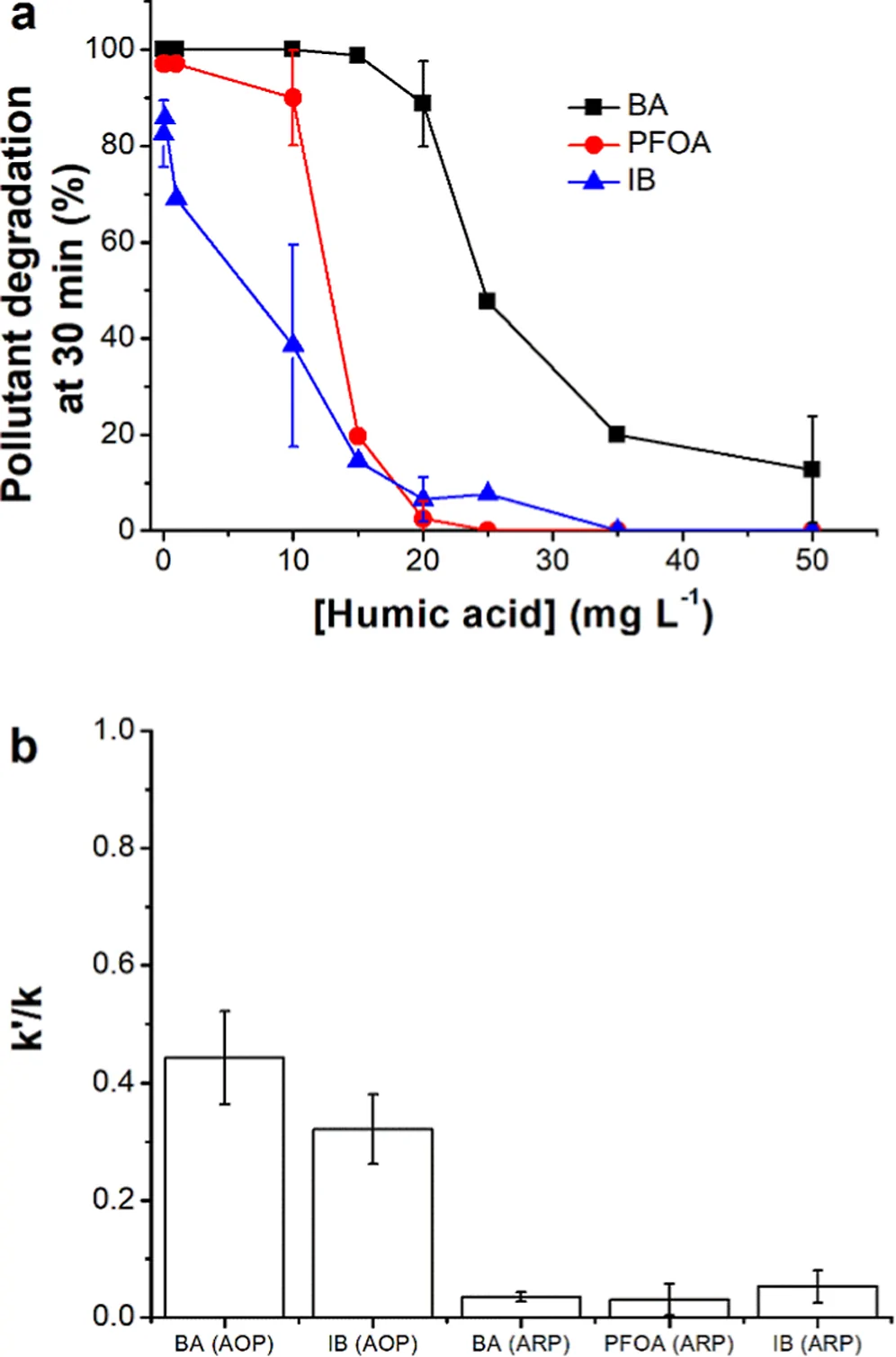
Role of humic acids in the studied processes. (a) Pollutant degradation percentages obtained after 30 min of irradiation during the UVC/SO_3_^2–^/I^–^ process as a function of the initial humic acid concentration. (b) Changes in pseudo-first-order kinetic rate constants for each contaminant during the UVC/H_2_O_2_ (AOP) and UVC/SO_3_^2–^/I^–^ (ARP); *k*′ and *k* correspond to the values obtained with and without 50 mg L^–1^ of humic acids, respectively. ARP working conditions: [SO_3_^2–^]_0_ = 10 mM, [I^–^]_0_ = 2 mM, pH_0_ = 12.0, N_2_ atmosphere. AOP working conditions: [H_2_O_2_]_0_ = 5 mM, pH_0_ = 7.0. In all cases, the starting solution contained 100 μM of BA, IB, and PFOA, respectively.

When changing humic acids with tryptophan (1 mg L^–1^ = 0.65 mgC L^–1^), to simulate the effect of protein-like substances,^76^ the inhibitory effect was considerably less marked (see Figure S10a,b). Even though no appreciable enhancement was observed, indole-containing compounds have been reported to be capable of photoproducing e_aq_^–^,^16,77^ as well as being considerably less reactive with e_aq_^–^ than benzoates (the tryptophan-e_aq_^–^ bimolecular rate constant is ca. 3 × 10^8^ M^–1^ s^–1^),^53^ explaining the observed lower hindering effect. For these reasons, although still an oversimplification, humic acids were selected as the representative probe for studying DOM effects because they are more likely than other DOM fractions to significantly hinder ARP performances.

Since 50 mg L^–1^ of humic acids (equivalent to 19 ± 2 mgC L^–1^, a concentration within the range present in concentrated streams)^30,42–44^ already caused significant e_aq_^–^ quenching, this concentration was used to compare performances between the UVC/SO_3_^2–^/I^–^ and the UVC/H_2_O_2_. As shown in Figure 5b, while the pollutant abatement rates by the ARP were diminished to >95%, the analogues with the AOP were 60–70%, indicating that the humic acids affected considerably more the reductive process than the oxidative one, in agreement with other works.^66,78–80^ Besides the e_aq_^–^ and HO^•^ quenching, DOM can also diminish the efficiency of AOPs and ARPs through a screening effect, reducing photon availability in the system. It should be borne in mind, however, that under aerobic conditions, illuminated DOM can also generate ROS capable of oxidizing, in this case, BA and IB, thereby partially compensating the aforementioned hindering effect.^81,82^

Since humic acids are macromolecules containing aromatic rings enriched with benzoate groups,^83^ these findings are consistent with the previously observed rapid BA degradation with ARPs. Moreover, as shown in Figure S11, the UVC/H_2_O_2_ led to significantly faster decolourization rates than the UVC/SO_3_^2–^/I^–^.

On the basis of these results, an oxidative pretreatment should increase the performance of a subsequent ARP (*vide infra*).

### 3.4 Treatment Trains

#### 3.4.1 ARP → AOP

Despite the fact that the studied ARPs led to fast PFOA removal and defluorination, no significant mineralization was observed (Figure 2d). It is therefore hypothesized that the application of an AOP after an ARP will mineralize the generated defluorinated byproducts.

Although the addition of I^–^ enhanced the UVC/SO_3_^2–^ process, it was not oxidized significantly (see Section 3.1). This is an inconvenience toward the coupling of UVC/SO_3_^2–^/I^–^ with UVC/H_2_O_2_, since: (*i*) the I^–^ will act as an HO^•^ scavenger and increment the H_2_O_2_ consumption (see Figure S12);^84^ (*ii*) differently from SO_3_^2–^, I^–^ is not easily oxidized by H_2_O_2_ at neutral pH, requiring highly acidic conditions to oxidize it into I_2_ ^85^ or applying several UVC/H_2_O_2_ processes, eventually leading to a likelihood of the treatment’s cost increment; and (*iii*) the presence of I^–^ could also lead to the formation of iodinated byproducts when employing AOPs, which have potential carcinogenicity.^86^ Due to the lack of practical sense, it was decided to avoid the use of I^–^ in the reductive pretreatment.

The ARP → AOP treatment train was applied as schematized in Figure 1: the pollutant mixture was treated with UVC/SO_3_^2–^ for 24 h to reach the maximum defluorination and then neutralized until pH 7.0 with H_2_SO_4_, and 10 mM of H_2_O_2_ was added (half of it oxidized the residual 4.5 ± 0.2 mM of SO_3_^2–^, see Figure 2b), remaining 5.0 ± 0.5 mM of H_2_O_2_ at disposal for the AOP. The UVC/H_2_O_2_ was then carried out for 4 h in an analogous way to that described in Section 3.2, adding a second dose of H_2_O_2_ (5 mM) after 2 h of treatment due to its consumption in this period.

In agreement with the work by Zhang et al.,^32^ the ARP pretreatment allowed the mineralization of PFOA, reaching a value of 91 ± 3% after 4 h (see Figure 6a), whereas for the AOP alone, the already discussed plateau at 60% mineralization was reached after 2 h of treatment; a second addition of H_2_O_2_ 5 mM did not decrease the TOC decay. However, a >99% TOC reduction was not reached, which could be related to the release of gaseous products or some recalcitrant PFASs in the ARP pretreatment as previously discussed (see Section 3.1).

**Figure 6:**
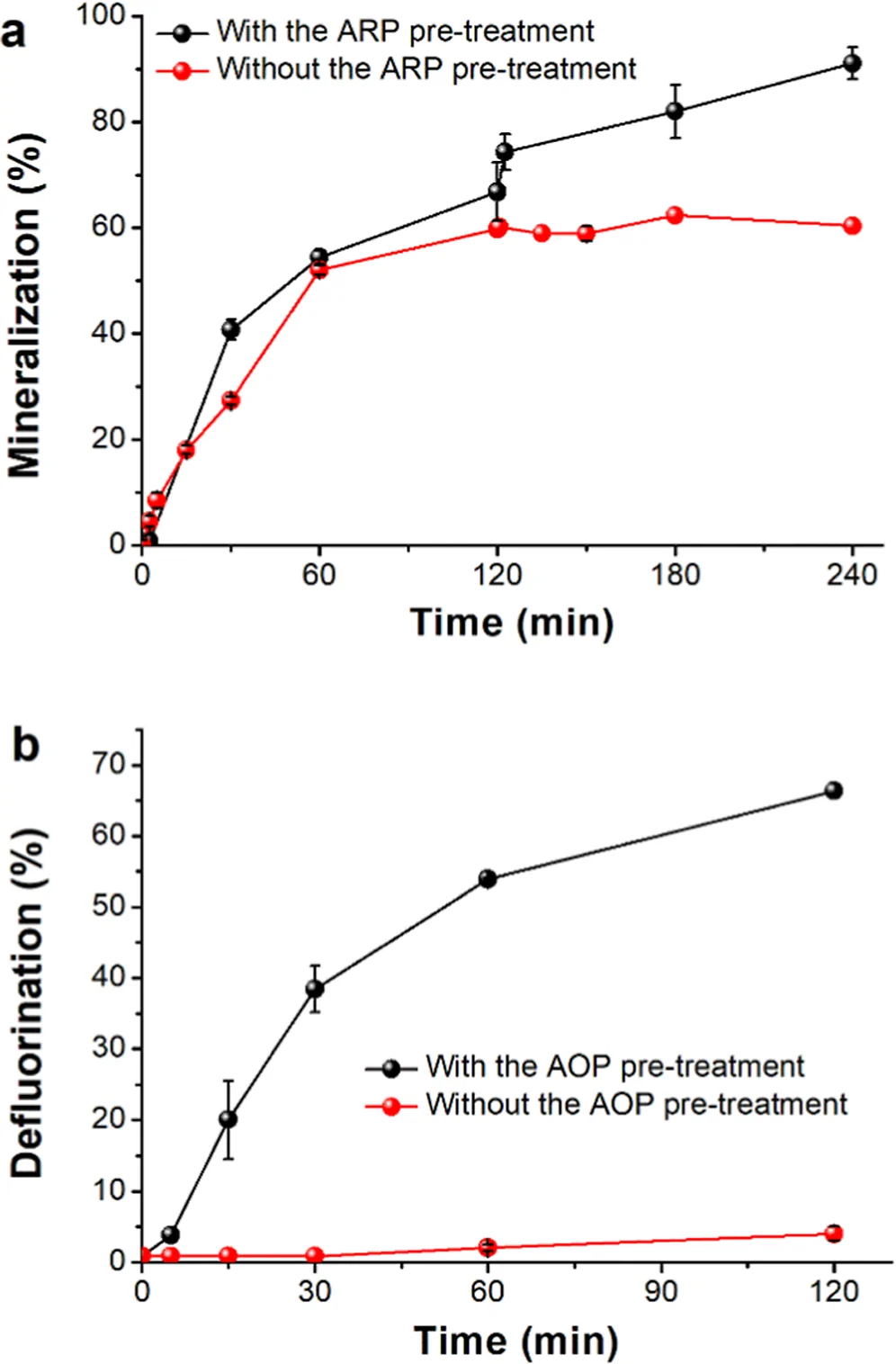
Results from treatment trains. (a) ARP → AOP: mineralization kinetics by UVC/H_2_O_2_ in ultrapure water with and without the pretreatment of UVC/SO_3_^2–^ for 24 h; H_2_O_2_ 5 mM was added at 0 and 120 min in both cases. (b) AOP → ARP: defluorination kinetics by UVC/SO_3_^2–^/I^–^ in a water matrix containing 50 mg L^–1^ of humic acids with and without the pretreatment of UVC/H_2_O_2_ of 2 h. ARPs were carried out under anaerobic conditions and pH = 12.0, whereas the UVC/H_2_O_2_ at aerobic conditions and pH = 7.0. In all cases, the starting solution contained 100 μM of BA, IB, and PFOA, respectively.

The differences between water treatment efficiencies by the AOP with and without the ARP pretreatment became more exacerbated when adding NO_2_^–^ (see later Section 3.5).

#### 3.4.2 AOP → ARP

The hypothesis for the AOP → ARP treatment train consists of the elimination of humic acids, which demonstrated to be strong e_aq_^–^ scavengers in Section 3.3.3. Therefore, if the DOM content from the influent is high, it is expected to obtain faster PFASs defluorination rates when employing the AOP → ARP treatment train than if applying the ARP alone.

The UVC/H_2_O_2_ pretreatment was applied for 2 h, a moment when, in ultra-pure water, BA and IB were already mineralized and the H_2_O_2_ concentration was negligible (see Figure 3b). At this point, it was possible to directly proceed with the ARP, adjusting the pH until 12.0, adding the SO_3_^2–^ 10 mM and I^–^ 2 mM, and bubbling N_2_ to achieve anoxic conditions as shown in Figure 1. Therefore, differently from the ARP → AOP approach, with this combination I^–^ can be used.

As anticipated, a notable PFOA defluorination enhancement was observed when employing the UVC/H_2_O_2_ pretreatment prior to the UVC/SO_3_^2–^/I^–^ process in a water matrix containing 50 mg L^–1^ of humic acids: while the ARP alone achieved negligible defluorination rates (4% in 2 h), the combined AOP → ARP treatment resulted in 66% defluorination in 2 h (see Figure 6b), demonstrating the usefulness of the proposed strategy, in line with the results exhibited in the work of Fennell et al.^30^

The aforementioned treatment train was also applied in the absence of humic acids, but no significant differences were observed when comparing it with the ARP alone (see Figure S13). These results indicate that the AOP → ARP strategy is mostly useful with solutions containing very high DOM content, and that BA and IB did not produce a significant hindering effect on the PFOA defluorination rates.

### 3.5 Performances on Simulated Retentate Wastewater with Pollutants in Low Concentrations

The studied processes were applied on SRWW with the pollutants spiked at 1 μM each to investigate their performance in more realistic conditions. As shown in Figure S14a–c, UVC/H_2_O_2_ led to a *k*_obs_ = 7.5 ± 0.4 × 10^–2^ min^–1^ for BA and a *k*_obs_ = 5.7 ± 0.2 × 10^–2^ min^–1^ for IB, while the degradation of PFOA was negligible. The TOC (initial value, 27 ± 3 mg L^–1^, see Table 1) remained constant for 2 h, indicating that the HO^•^ were significantly scavenged by the NO_2_^–^.

When employing the UVC/SO_3_^2–^/I^–^, negligible BA and IB removals were obtained, while appreciable PFOA removal was achieved (*k*_obs_ = 3.6 ± 0.7 × 10^–3^ min^–1^). As expected, slower degradations were obtained for the ARP without the I^–^, obtaining a PFOA *k*_obs_ of 1.7 ± 0.1 × 10^–3^ min^–1^. Except for the scarce degradation of BA, these results correlated well with those obtained with 100 μM of pollutant concentration in ultrapure water and the individual effects of main interferences: humic acids, NO_2_^–^, and NO_3_^–^. For the BA, with such low concentrations, it seems that the e_aq_^–^ react predominantly with the humic acids.

To enhance the performance of the oxidative process, NO_2_^–^ was removed with the UVC/SO_3_^2–^ pretreatment as discussed previously. As shown in Figure S14d, 6 h were required to get rid of the 1.4 mM of NO_3_^–^ and the 0.6 mM of NO_2_^–^ in the SRWW. When the UVC/H_2_O_2_ was subsequently applied, fast DOM mineralization was achieved (45% mineralization within 2 h), which was not observed when this treatment was used alone (see Figure S14e). These results indicate that under simultaneous high NO_2_^–^ and DOM content, NO_2_^–^ remains the primary interference, the ARP → AOP sequence being more effective than the AOP → ARP one. In fact, very similar degradation profiles were observed for the PFOA when the oxidative pretreatment was or was not used (see Figure S14f).

## 4 Conclusions

This work provides valuable insights into the interplay between ARPs and AOPs, especially for the treatment of matrices containing high DOM, cation, and anion concentrations. Results demonstrated that UVC/H_2_O_2_ pretreatment restored the UVC/SO_3_^2–^/I^–^ efficiency under high humic acid concentrations, while in the inverse approach, the UVC/SO_3_^2–^ pretreatment allowed the PFOA mineralization by the subsequent UVC/H_2_O_2_ process. These findings offer practical guidance for designing integrated PFASs treatment strategies. Both configurations (ARP → AOP or AOP → ARP) presented advantages and drawbacks:

- ARP → AOP (UVC/SO_3_^2–^ → UVC/H_2_O_2_): It is useful in wastewater containing high NO_2_^–^ concentrations (preferably with low DOM content) and/or seeking PFASs mineralization. Long treatments are expected due to the compatibility with the less efficient UVC/SO_3_^2–^ process. Special attention should be paid to high Cl^–^ concentrations, since the AOP post-treatment might lead to the release of chlorinated byproducts in the effluent.
- AOP → ARP (UVC/H_2_O_2_ → UVC/SO_3_^2–^/I^–^): It is useful in wastewater containing high DOM concentrations but low NO_2_^–^ concentrations and/or seeking faster PFASs defluorination rates. Additional advantages could be the absence of NO_3_^–^ in the resulting effluent due to its removal in the ARP post-treatment. A similar removal is hypothesized for chlorinated byproducts generated during the oxidative step in the presence of high Cl^–^ concentrations. As drawbacks, this approach leads to the release of PFASs-defluorinated byproducts that require further management and evaluation. Besides, the formation of iodinated byproducts (although not detected in this work) cannot be disregarded.

When treating the SRWW, the most efficient treatment train was the ARP → AOP one. However, results suggested that the UVC/SO_3_^2–^ pretreatment should last, at least, 6 h for the subsequent oxidative process to be efficient, expecting high electrical consumption (*E*_EO_ > 1 × 10^4^ kW h m^–3^). However, the employed setup might not be energy-efficient enough; the obtained *E*_EO_ could be easily improved with a better reactor design and use of available UVC-LEDs instead of classical Hg low-pressure lamps.

This work also leaves several open questions that warrant further investigation. On the one hand, life cycle assessments and cost analyses should be conducted to evaluate the feasibility of applying these treatment trains to the aforementioned concentrates. In this context, the abrupt pH adjustments employed in this study (from 7.0 to 12.0 and *vice versa*) should also be reconsidered, exploring intermediate operating conditions (e.g., pH 9–10) as a potentially more practical and sustainable alternative, avoiding pH changes. Another strategy is employing the AOPs that are efficient at pH 12 (e.g., ozonation). As demonstrated in this work, the effect of cations might affect the ARPs (case of Ca^2+^, forming CaSO_3_, which was able to efficiently degrade the pollutant mixture) or the defluorination measurements (case of Mg^2+^ through MgF_2_ precipitation). Future studies might also bear in mind the presence of transition metals, which were not considered in this study, since they are usually present at considerably lower concentrations than Ca^2+^ or Mg^2+^, as well as the fact that they shall likely be in the form of insoluble (oxy)­hydroxides at the working pH (alkaline) conditions. Transition metals might scavenge e_aq_^–^ but at the same time can catalyze the H_2_O_2_ decomposition, which might also accelerate the AOP. Finally, if high Cl^–^ concentrations are present, although we did not observe a relevant hindering in the studied processes, the plausible formation of chlorinated byproducts by AOPs should be taken into consideration (an analogous rationale is applied for high Br^–^ concentrations).

## Supplementary Material



## Notes

Employed reagents, investigated and detected photoproducts by HRMS, DoE-RSM of UVC/SO_3_^2–^/I^–^, effect of O_2_ and H^+^ on UVC/SO_3_^2–^/I^–^, UVC/H_2_O_2_ preliminary results, pollutants photolysis at pH 7 and aerobic conditions, effect of anions on UVC/H_2_O_2_ process, effect of anions on UVC/SO_3_^2–^/I^–^ process, pollutants photolysis in the presence of NO_2_^–^ and NO_3_^–^, including also their own, TN and SO_3_^2–^ concentration decays during UVC/SO_3_^2–^/I^–^ process with NO_2_^–^ and NO_3_^–^, effect of Ca^2+^ and Mg^2+^ on the UVC/SO_3_^2–^/I^–^ process, effect of humic acids and tryptophan on the UVC/SO_3_^2–^/I^–^ process, humic acid decolourization kinetics by UVC/H_2_O_2_ and UVC/SO_3_^2–^/I^–^, effect of I^–^ 2 mM on the UVC/H_2_O_2_ process, AOP→ARP treatment train in ultrapure water, results obtained with SRWW, and mathematical deduction for the estimation of steady-state concentrations of e_aq_^–^ and HO^•^, respectively, in the presence of NO_2_^–^ 1 mM (PDF)
