# Supplementary material for: Better Together Than Apart: Degradation of Emerging Contaminants in Concentrated Streams Using UV/Sulfite/Iodide and UV/Hydrogen Peroxide Treatment Trains
Source: ACS Environ Au. 2026 Jul 1;6(5):890–902. doi: 10.1021/acsenvironau.6c00098 (PMC13602341; doi:10.1021/acsenvironau.6c00098)
Supplement: Supplementary file 1 [file vg6c00098_si_001.pdf]

Supporting information

Better together than apart: degradation of emerging contaminants in concentrated streams using UV/sulfite/iodide and UV/hydrogen peroxide treatment trains

Iván Sciscenko,[1,*] Agustín París-Reche,[2,3] Ana Agüera,[2,3] Patricia Plaza-Bolaños,[2,3] Claudio Minero,[1] and Marco Minella[1]

(1) University of Turin, Department of Chemistry, Via Pietro Giuria 7, Turin 10125, Italy.
(2) Department of Chemistry and Physics, University of Almeria, Carretera de Sacramento s/n, Almería 04120, Spain.
(3) Solar Energy Research Centre (CIESOL), Joint Centre of the University of Almeria-CIEMAT, Carretera de Sacramento s/n, Almería 04120, Spain.

Correspondence: ivanmatias.sciscenko@unito.it

Number of pages: 13

Number of tables: 2

Number of figures: 14

Number of text sections: 1

Table S1. Reagents' list.

**Reagent**	**Manufacturer**
Perfluorooctanoic acid (PFOA, > 96%)	Thermo Scientific
Benzoic acid (BA, > 99%)	Sigma Aldrich
Ibuprofen sodium salt (IB, > 99%)	Sigma Aldrich
EDTA (ethylenediaminetetraacetic acid, >99%)	Sigma Aldrich
Humic acid sodium salt (technical grade)	Sigma Aldrich
Na_2SO_3 (≥ 98%)	Sigma Aldrich
$NaIO_3$ (≥ 98%)	Sigma Aldrich
KI (≥ 98%)	VWR Chemicals
Methanol (UHPLC grade)	VWR Chemicals
Acetonitrile (UHPLC grade)	VWR Chemicals
H_2O_2 (33% m/V)	VWR Chemicals
H_2SO_4 (98%)	VWR Chemicals
H_3PO_4 (85%)	VWR Chemicals
Phenol (>99%)	VWR Chemicals
$CaCl_2$ (>95%)	Merck
$MgCl_2$•$6H_2O$ (>99%)	Merck
4-aminoantipyridine	Merck
Peroxidase horseradish	Merck
NaOH (≥ 98%)	Merck
NaF (≥ 98%)	Merck
NaCl (≥ 98%)	Merck
Na_2SO_4 (≥ 98%)	Merck
$NaNO_3$ (≥ 98%)	Merck
$NaNO_2$ (≥ 98%)	Merck
NaH_2PO_4•H_2O (≥ 98%)	Alfa Aesar
Na_3PO_4 (≥ 98%)	Alfa Aesar
$NaHCO_3$ (≥ 98%)	Alfa Aesar
Borax = $Na_2B_4O_7$•$10H_2O$ (≥ 98%)	Alfa Aesar
Na_2CO_3•$10H_2O$ (≥ 98%)	Alfa Aesar
K_2CO_3 (≥ 98%)	Alfa Aesar
Tryptophan (>99%)	Acros Organics

Table S2. Photoproducts investigated and detected (**in bold**) during the degradation of PFOA 100 µM by UVC/SO_3^{2-}/I^- at optimal conditions.

Compound	Molecular formula	Theoretical $[M-H]^-$ (*m/z*)	Error (ppm)	Structure $[M-H]^-$	Ref.
**PFOA**	**$C_8HF_{15}O_2$**	**412.9664**	**-2.5**	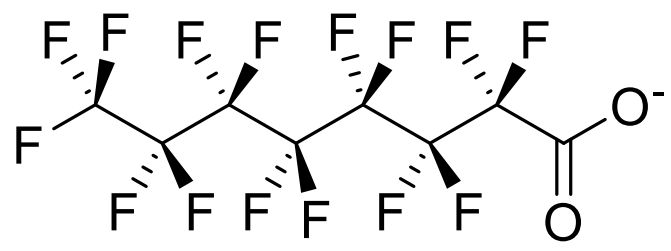	[1,2]
TP1	$C_8H_2F_{13}O_3$	392.9801	-	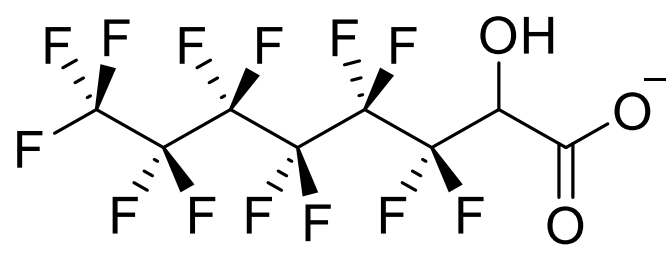	[1,3]
TP2	$C_7HF_{15}O$	384.9715	-	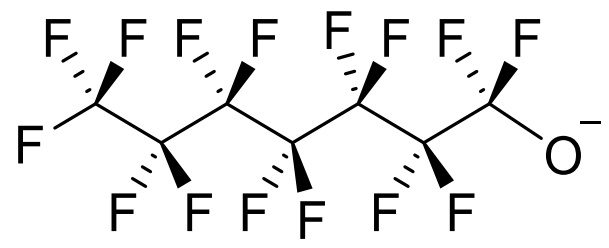	[1–4]

**TP3 (PFHpA)**	**$C_7HF_{13}O_2$**	**362.9696**	**-1.5**	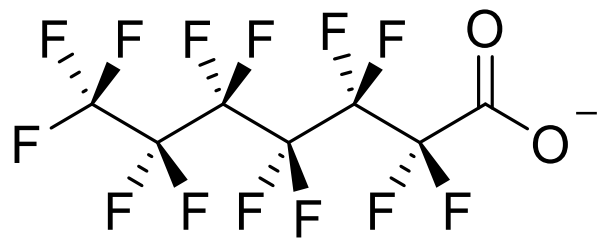	1–3
TP4 (PFHxA)	$C_6HF_{11}O_2$	312.9728	-	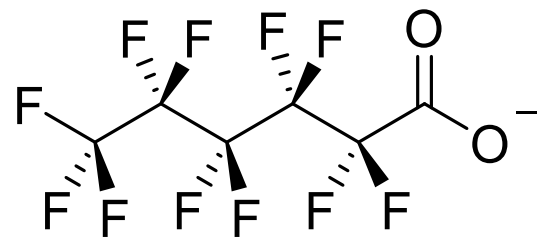	1,3
**TP5 (PFPeA)**	**$C_5HF_9O_2$**	**262.9760**	**0.1**	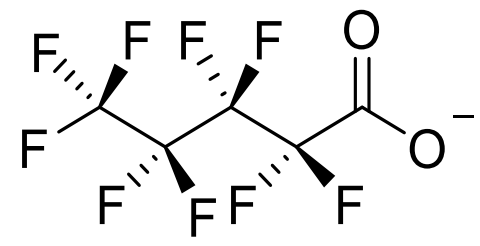	
**TP6 (PFBA)**	**$C_4HF_7O_2$**	**212.9792**	**0.6**	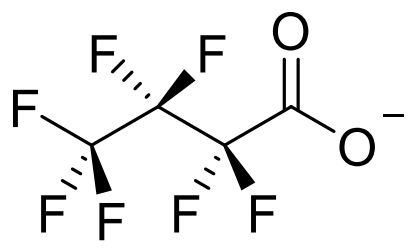	
**TP7**	**$C_8H_2F_{14}O_2$**	**394.9758**	**3.2**	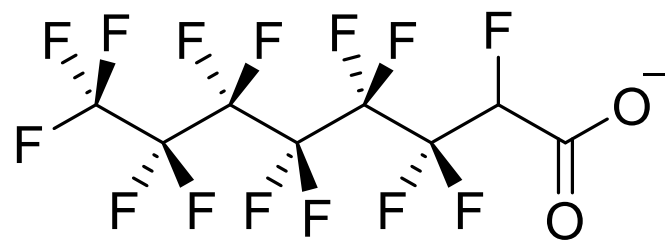	1–4
**TP8 (PFHxS)**	**$C_6HF_{13}O_3S$**	**398.9366**	**-2.8**	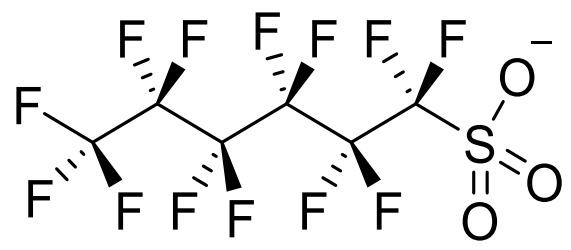	1
TP9 (PFHpS)	$C_7HF_{15}O_3S$	448.9334	-	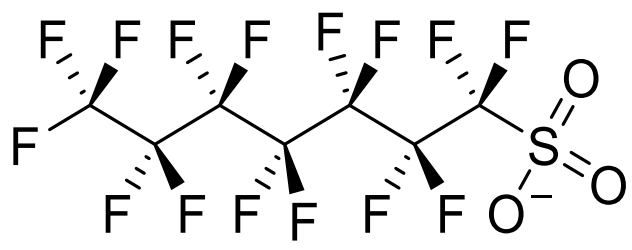	
**TP10 (FHxEA)**	**$C_8H_3F_{13}O_2$**	**376.9852**	**-0.8**	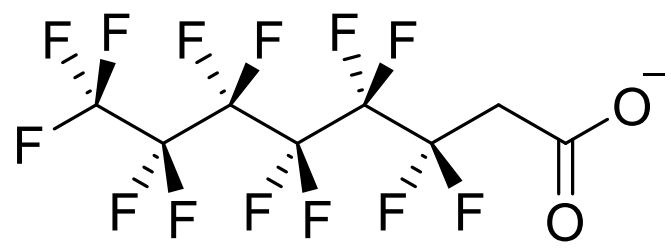	1–4
TP11	$C_7H_3F_{13}O_3S$	412.9522	-	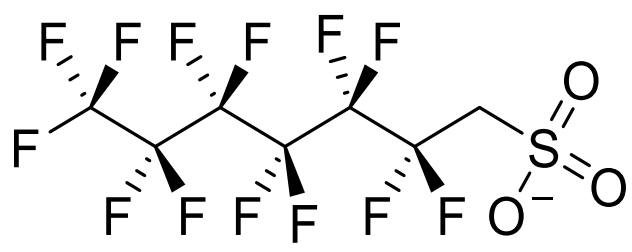	1,2
**TP12A**	**$C_8H_2F_{14}O_5S$**	**474.9326**	**-4.1**	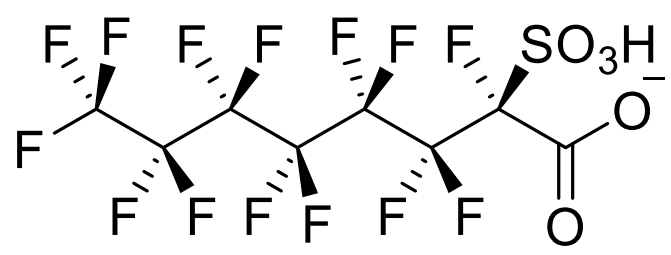	
**TP12B**			**-4.3**	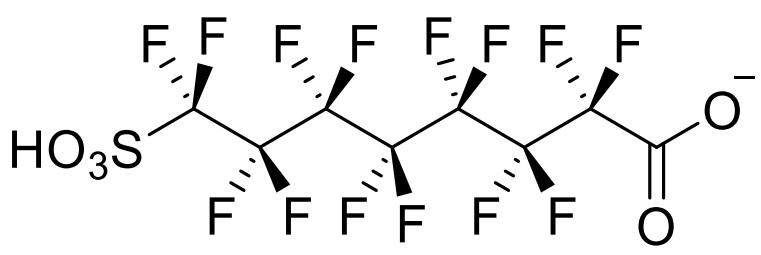	
**TP13**	**$C_7HF_{13}O_3S$**	**410.9366**	**-1.2**	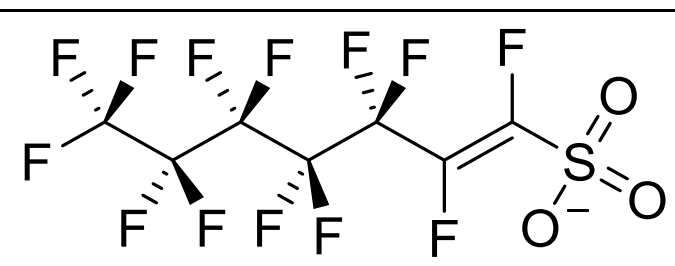	
**TP14**	**$C_8H_3F_{13}O_5S$**	**456.9420**	**0.4**	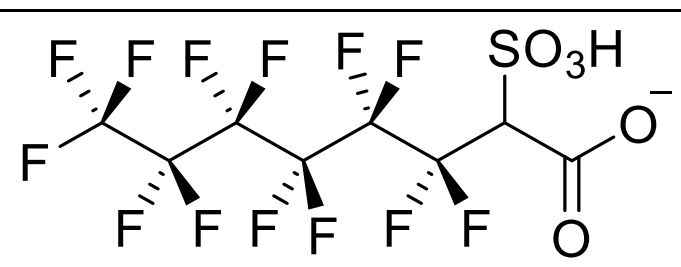	
**TP15A**	**$C_7H_2F_{14}O_3S$**	**430.9428**	**-5.4**	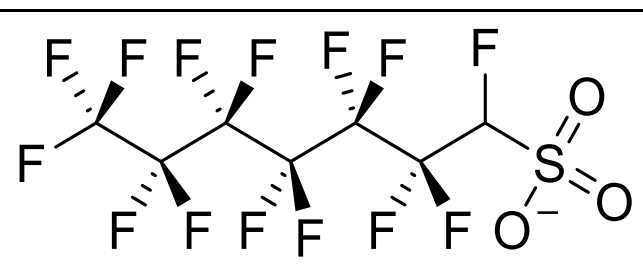	

**TP15B**			**-3.8**	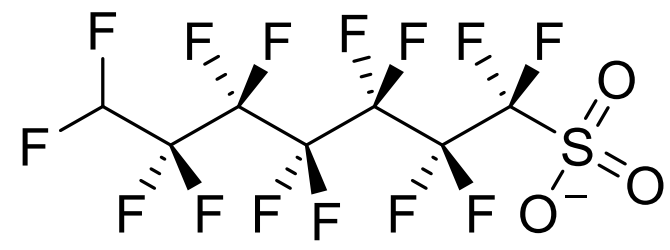	
**TP16**	**$C_7H_2F_{12}O_3S$**	**392.4603**	**1.5**	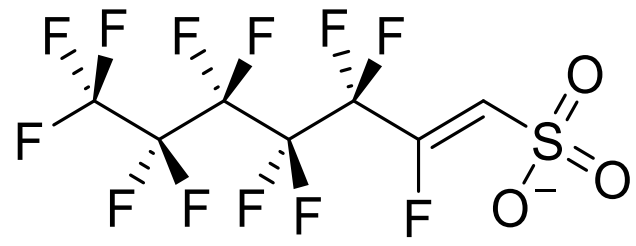	
**TP17**	**$C_8H_2F_{12}O_5S$**	**436.9358**	**0.1**	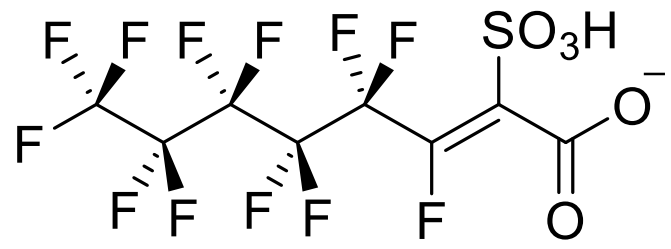	
**TP18**	**$C_8H_4F_{12}O_5S$**	**438.9515**	**0.2**	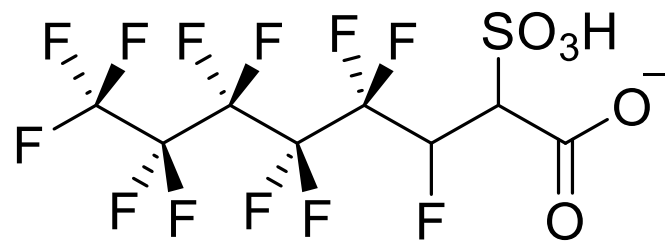	
TP19	$C_9H_4F_{12}O_8S$	498.9362	-	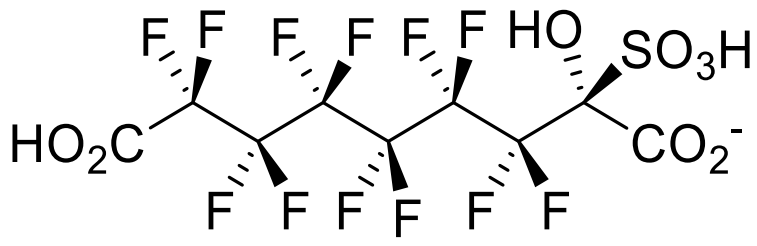	1
**TP20 (PFPeS)**	**$C_5HF_{11}O_3S$**	**348.9398**	**-0.8**	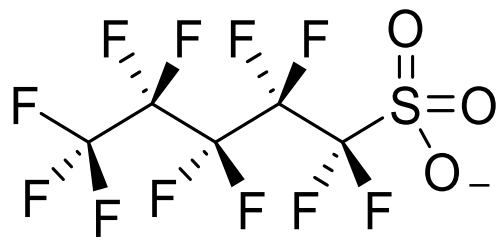	
TP21	$C_8H_4F_{12}O_7S$	470.9413	-	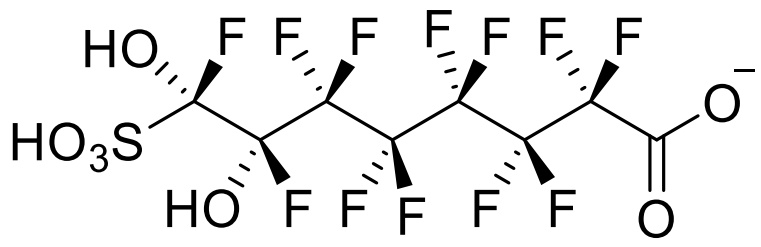	4
**TP22**	**$C_8HF_{13}O_2$**	**374.9696**	**-0.6**	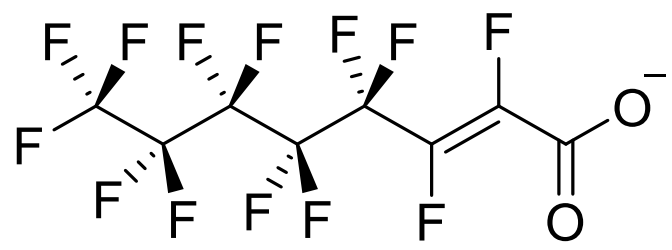	
TP23	$C_7H_2F_{12}O_3$	360.9739	-	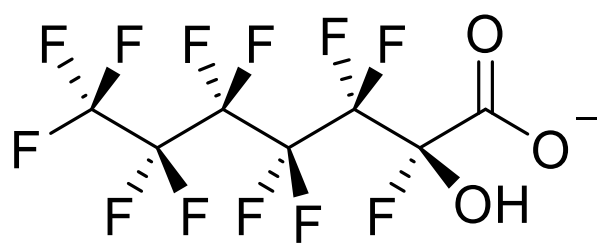	
TP24	$C_7H_3F_{11}O_3$	342.9833	-	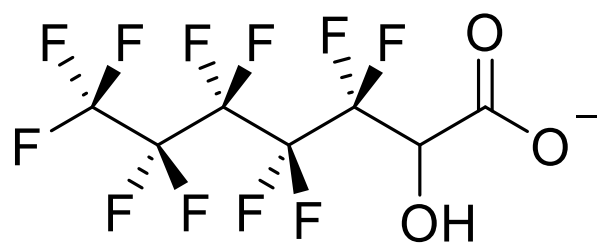	
TP25	$C_6H_2F_{10}O_2$	294.98223	-	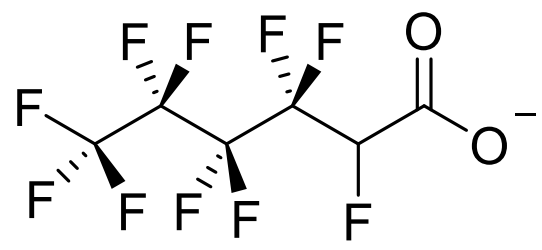	3,4
TP26	$C_6H_3F_9O_2$	276.9916	-	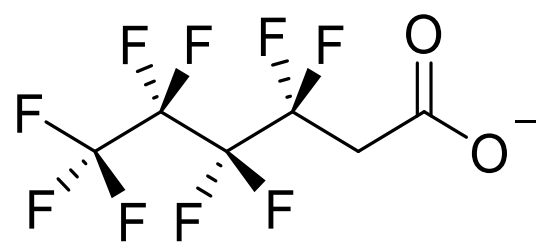	
TP27	$C_5H_2F_8O_2$	244.9854	-	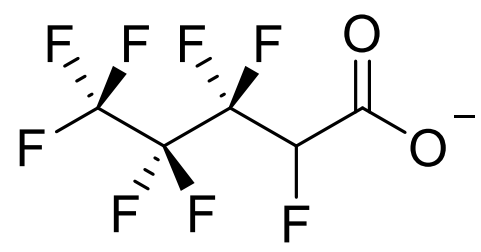	
TP28	$C_5H_3F_7O_2$	225.9864	-	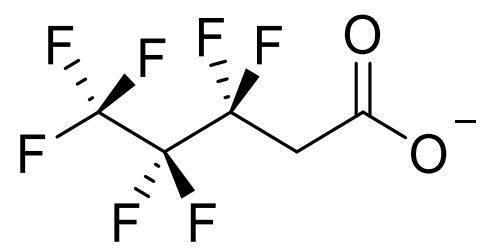	

TP29	$C_4H_2F_6O_2$	194.9886	-	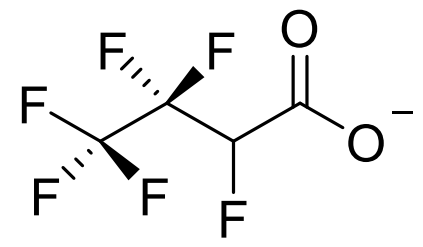	
TP30	$C_4H_3F_5O_2$	176.9980	-	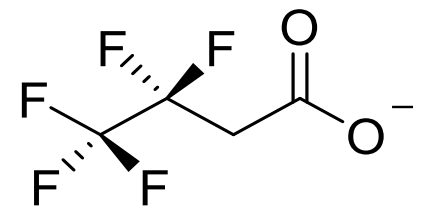	
TP31 (FPrA)	$C_3HF_5O_2$	162.9823	-	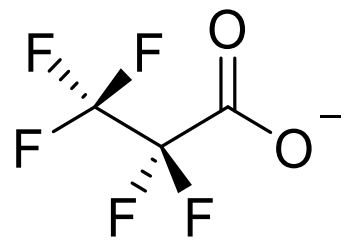	
TP32	$C_3H_2F_4O_2$	144.9918	-	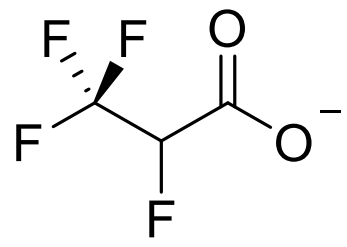	
TP33	$C_3H_3F_3O_2$	127.0012	-	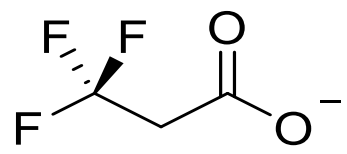	
TP34 (TFA)	$C_2HF_3O_2$	112.9855	-	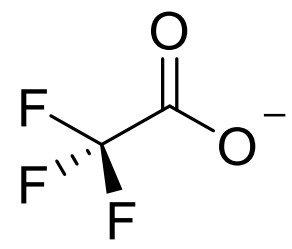	
TP35	$C_8HF_{14}O_2$	393.9680	-	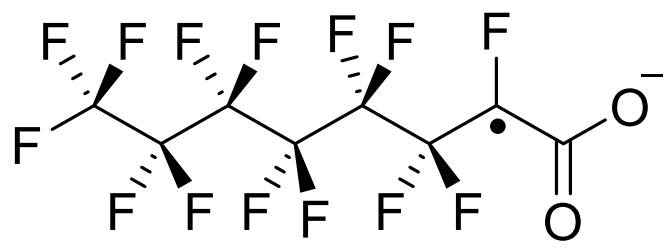	

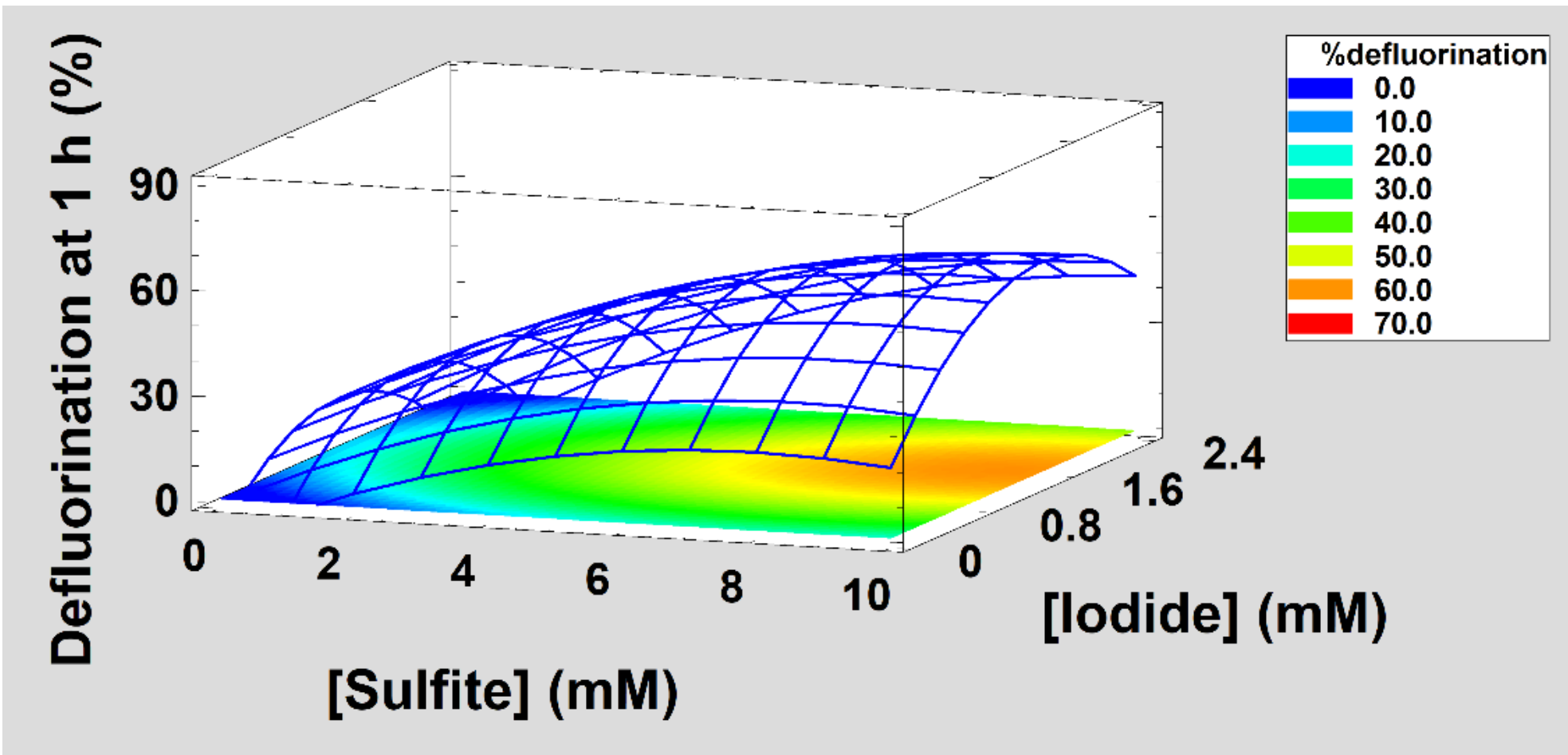

Figure S1. Design of Experiment-Response Surface Methodology results for the optimization of PFOA 100 µM degradation by UVC/SO_3^{2-}/I^- process at pH_0 = 12.0. Optimal conditions: [SO_3^{2-}] = 9.5 mM and [I^-] = 1.5 mM (R^2 = 0.78).

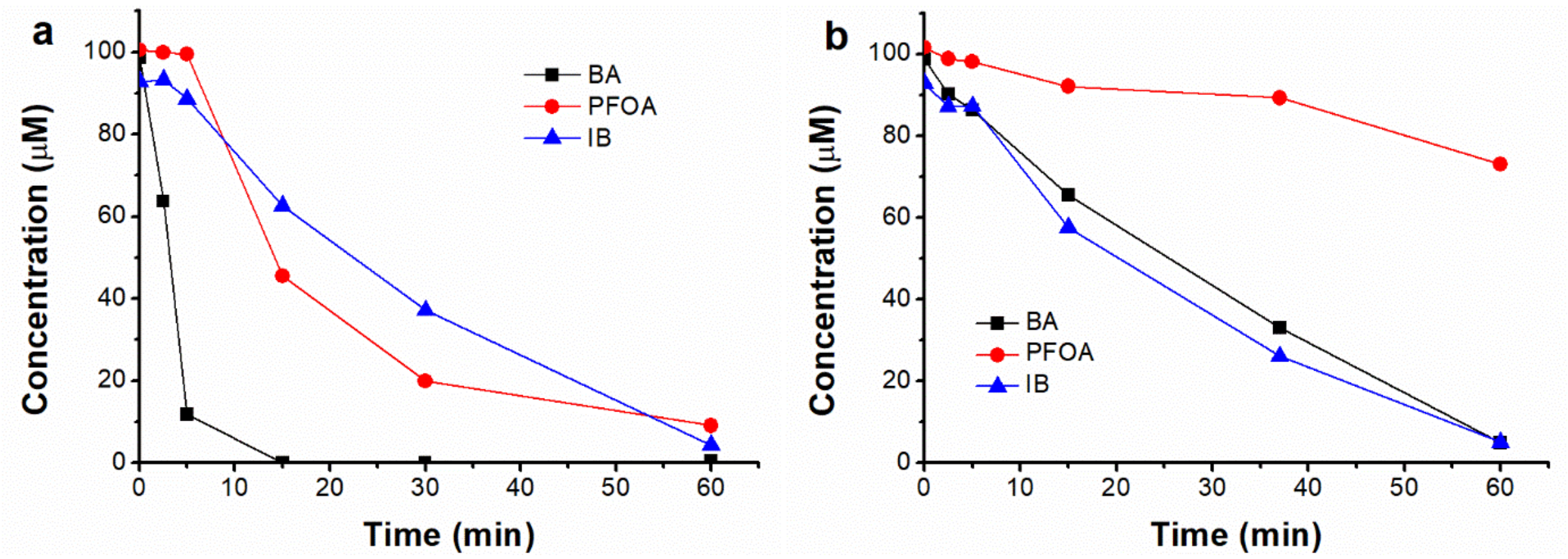

Figure S2. ARP preliminary tests results: pollutants removal by UVC/SO_3^{2-}/I^- at a) pH_0 = 12.0 and aerobic conditions, and b) pH_0 = 7.0 and anaerobic conditions. In both cases, $[SO_3^{2-}]_0$ = 10 mM, $[I^-]_0$ = 2 mM.

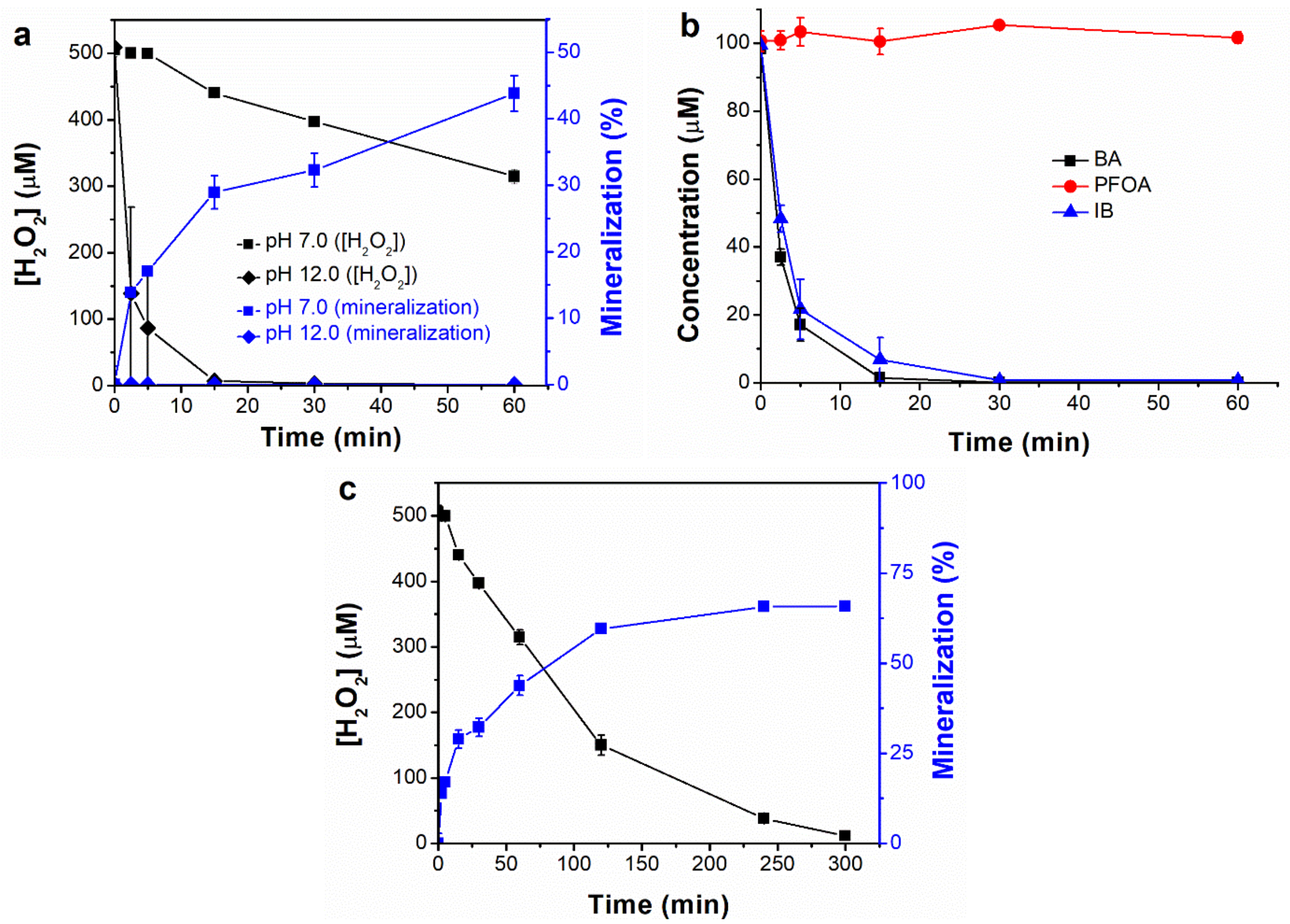

Figure S3. AOP (UVC/H_2O_2) preliminary tests experiments, $[H_2O_2]_0$ = 0.5 mM and aerobic atmosphere: a) alkaline vs. neutral pH on H_2O_2 consumption and pollutant mineralization, b) pollutants concentration decay (pH 7.0), and c) H_2O_2 consumption and produced mineralization (pH 7.0) for 300 min.

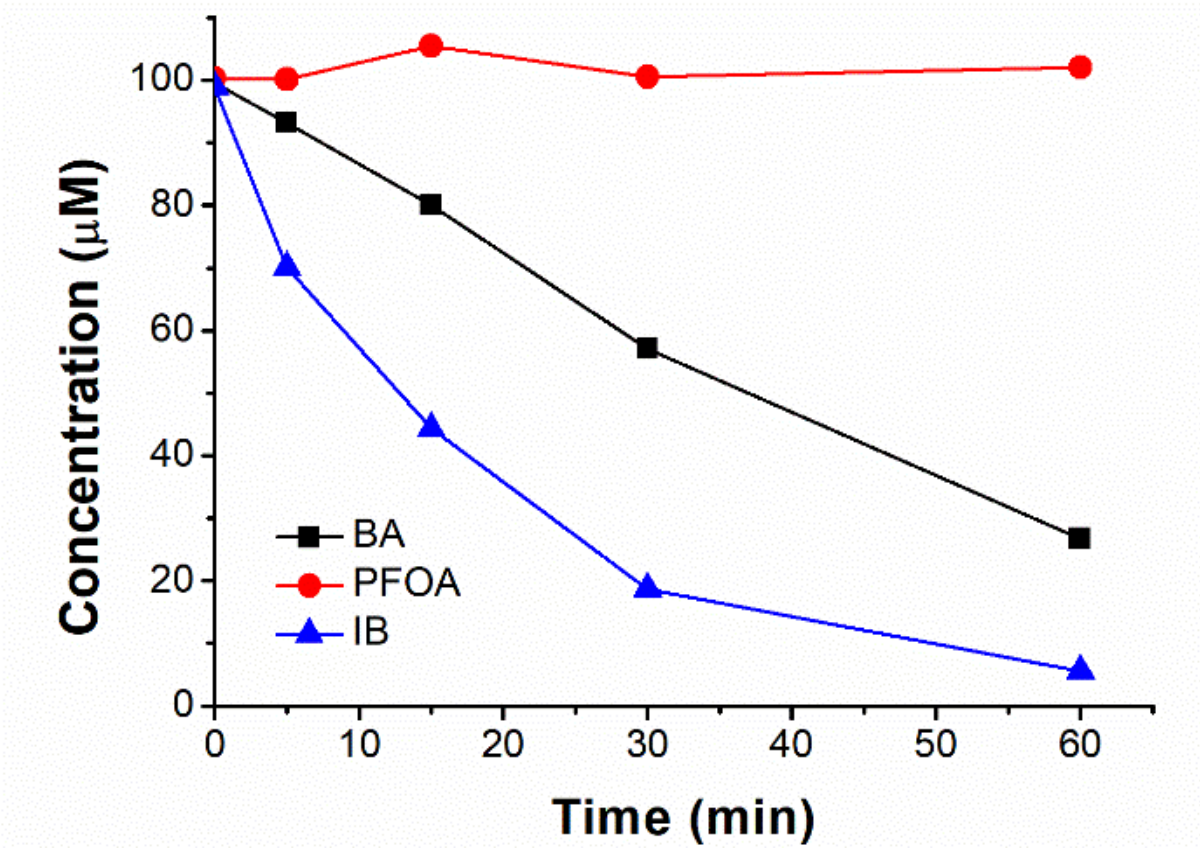

Figure S4. Pollutants photodegradation results by UVC light obtained at pH 7.0 and aerobic conditions.

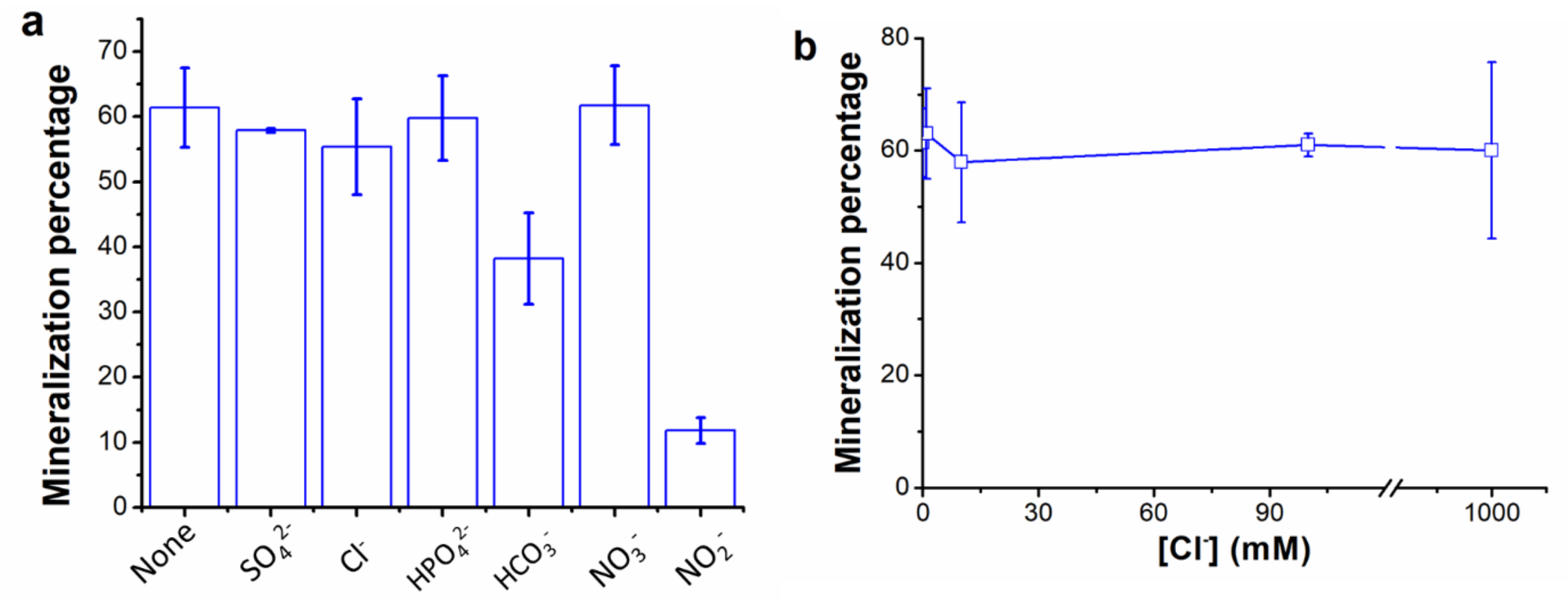

Figure S5. Effect of anions on the UVC/H_2O_2 process. Measured pollutants mineralization percentage after 1 h treatment in: a) the presence of 1 mM of specified anions, and b) with increasing Cl^- concentration. Conditions: ultra-pure water adjusted at $pH_0 = 7.0$, aerobic atmosphere, $[H_2O_2]_0 = 5$ mM, $[BA]_0 = [IB]_0 = [PFOA]_0 = 100$ μM.

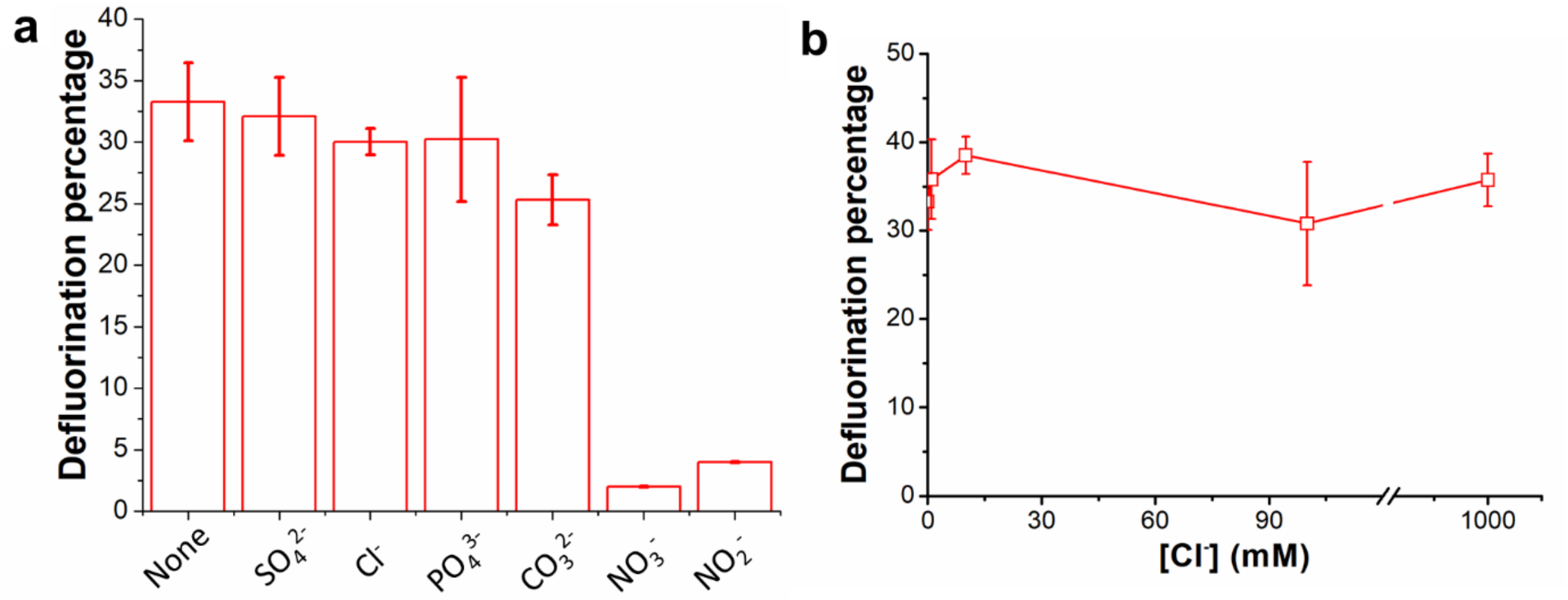

Figure S6. Effect of anions on the UVC/SO_3^{2-}/I^- process: a) PFOA defluorination percentage at 30 min by UVC/SO_3^{2-}/I^- in the presence of 1 mM of specified anions, and b) with increasing Cl^- concentration, respectively. Conditions: ultra-pure water adjusted at pH 12.0, N_2 atmosphere, $[SO_3^{2-}]_0 = 10$ mM, $[I^-]_0 = 2$ mM, $[BA]_0 = [IB]_0 = [PFOA]_0 = 100$ μM.

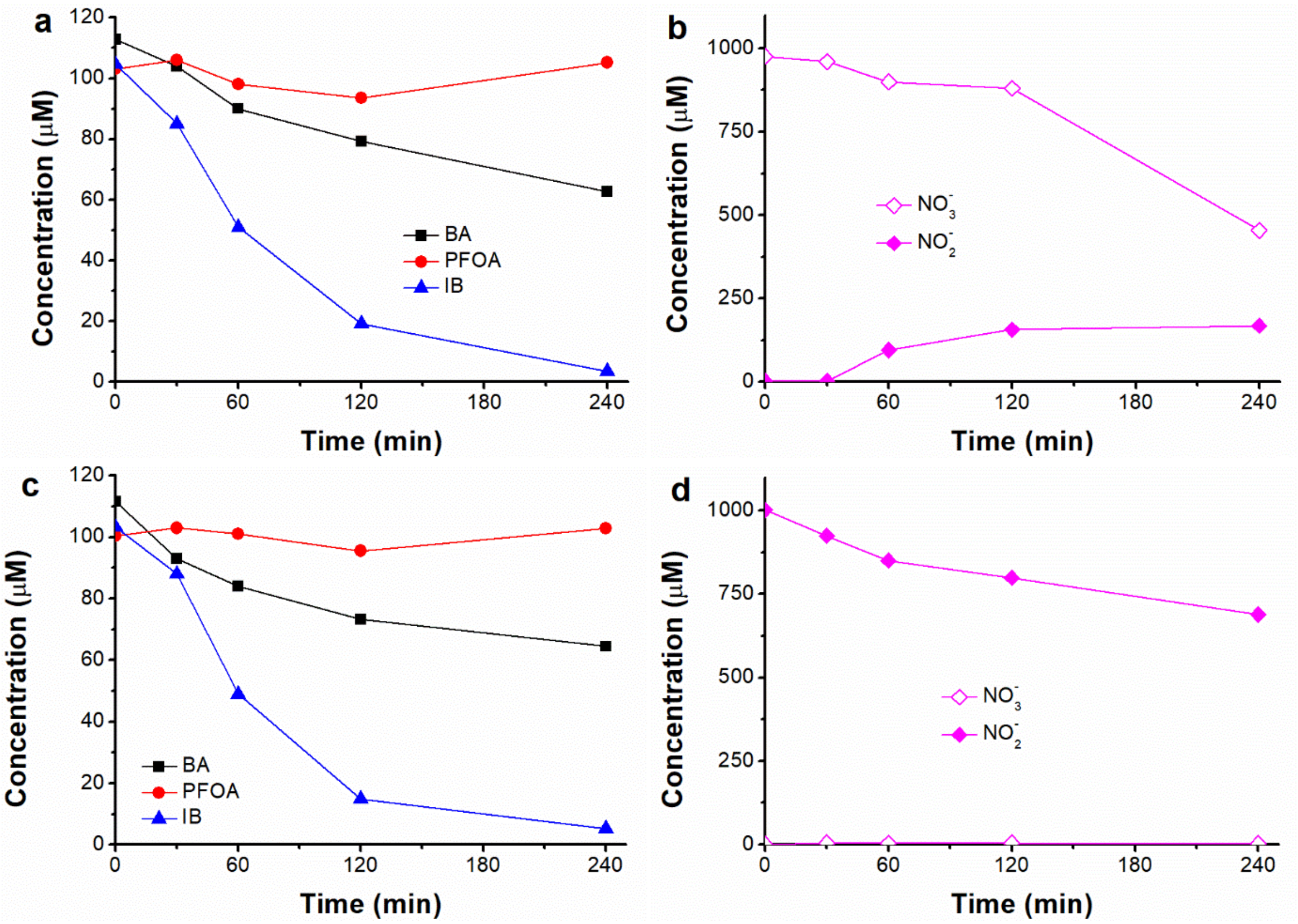

Figure S7. Pollutants photodegradation results by UVC light obtained in the presence of 1 mM of NO_3^- or NO_2^-, anaerobic conditions, and initial pH 12: a-b) results when adding NO_3^- and c-d) results when adding NO_2^-.

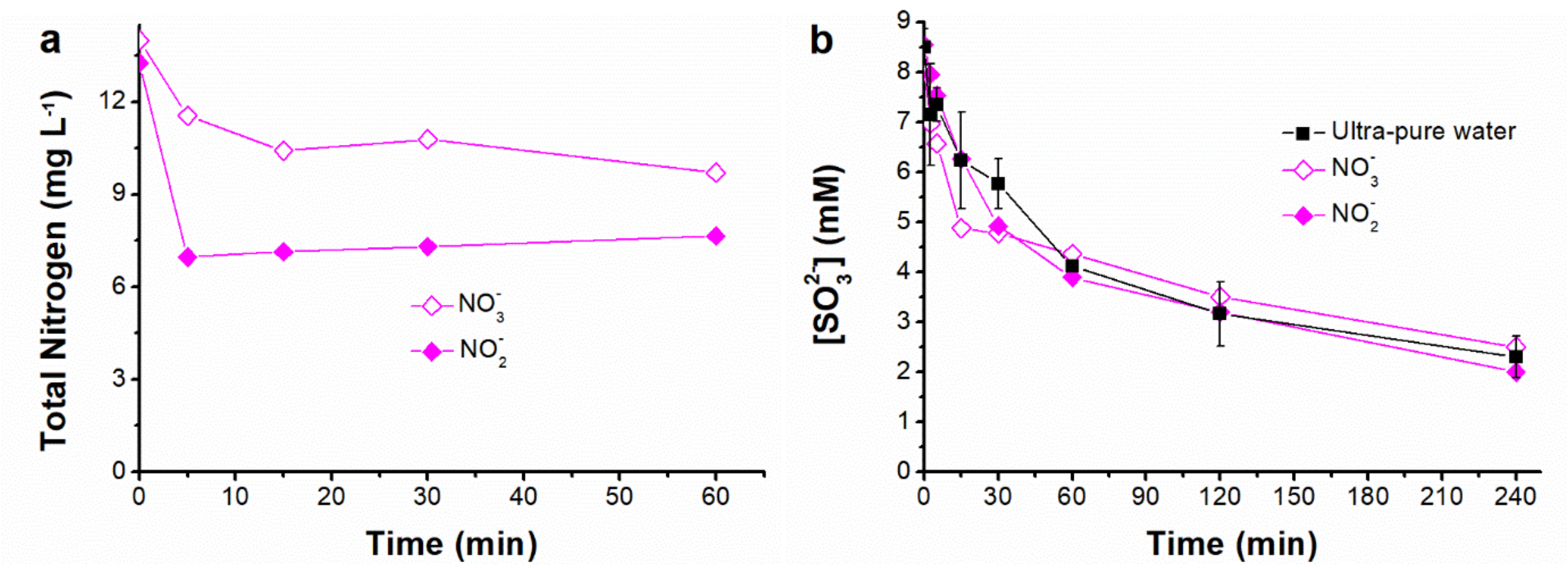

Figure S8. UVC/SO_3^{2-}/I^- applied in the presence of NO_2^- or NO_3^-, each spiked in 1 mM concentration: a) TN and b) sulphite concentration decays obtained in the presence of NO_3^- or NO_2^- 1 mM, respectively. Conditions: $[SO_3^{2-}]_0$ = 10 mM, $[I^-]_0$ = 2 mM, pH_0 = 12.0, N_2 atmosphere.

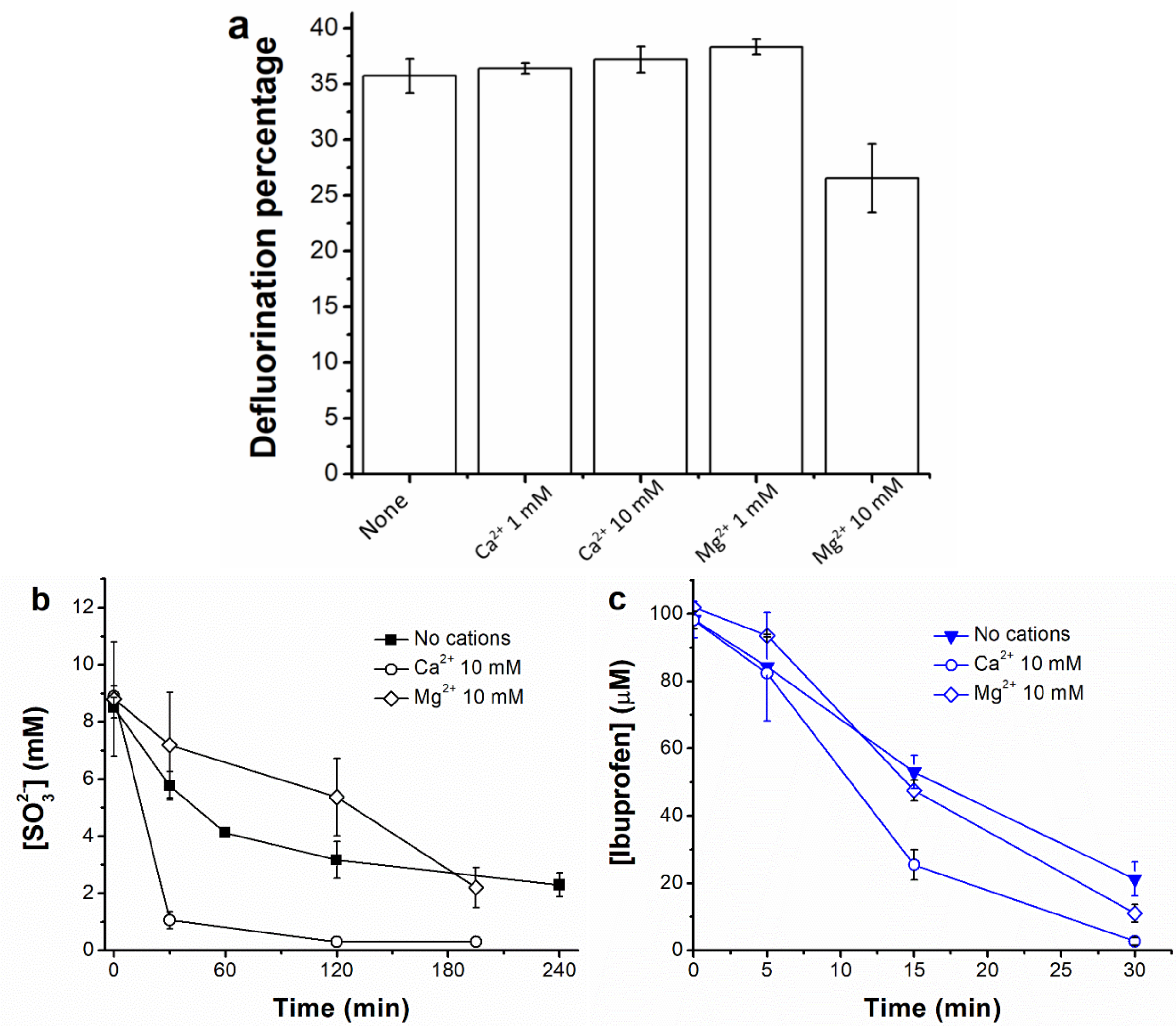

Figure S9. Effect of Ca^{2+} and Mg^{2+} on the UVC/SO_3^{2-}/I^- process: a) defluorination percentage obtained after 30 min of irradiation, b) SO_3^{2-} concentration kinetics, and c) ibuprofen degradation kinetics (benzoate and PFOA kinetics were not included as they were analogous to the one in ultra-pure water, the effect of cations not being appreciated). Conditions: $[Ca^{2+}]$ = 1 or 10 mM, $[Mg^{2+}]$ = 1 or 10 mM, $[SO_3^{2-}]_0$ = 10 mM, $[I^-]_0$ = 2 mM, pH_0 = 12.0, N_2 atmosphere.

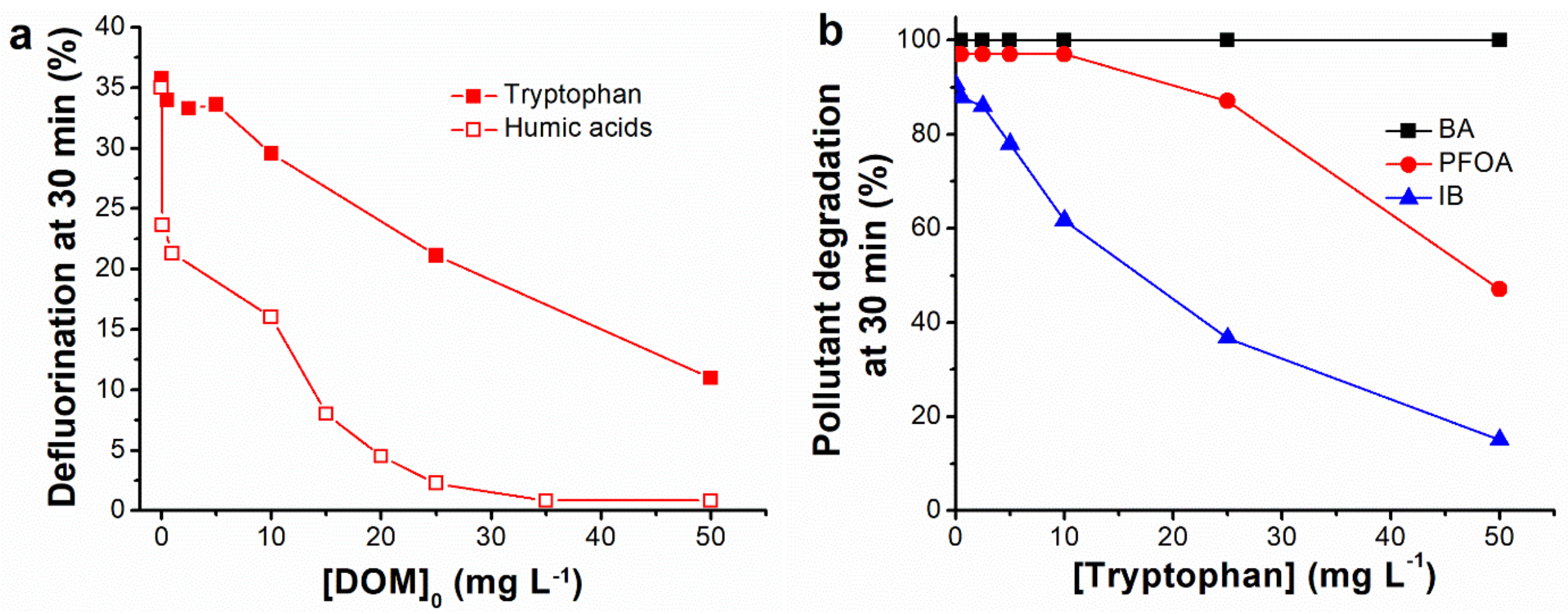

Figure S10. Effect of dissolved organic matter (DOM) on the UVC/SO_3^{2-}/I^- process: a) PFOA defluorination percentage obtained after 30 min of irradiation with different initial concentrations of

tryptophan and humic acids, and b) pollutants obtained degradation after 30 min of treatment with increasing concentration of tryptophan.

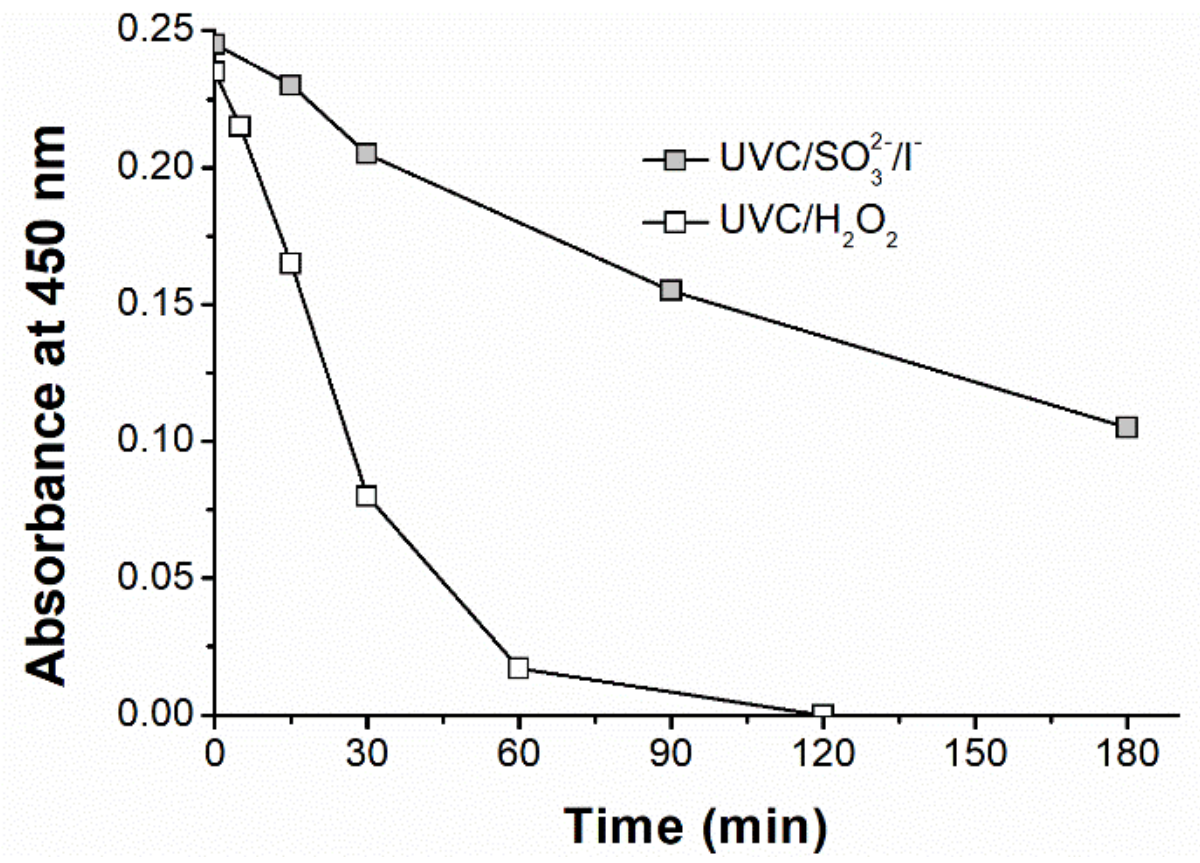

Figure S11. Humic acids absorbance decay at 450 nm (region where only humic acid absorbed light) by UVC/H_2O_2 and UVC/SO_3^{2-}/I^-. Conditions: AOP, $[H_2O_2]_0 = 5$ mM, $pH_0 = 7.0$; ARP, $[SO_3^{2-}]_0 = 10$ mM, $[I^-]_0 = 2$ mM, $pH_0 = 12.0$, N_2 atmosphere. Absorbance spectra measured at pH 7.0 in both cases.

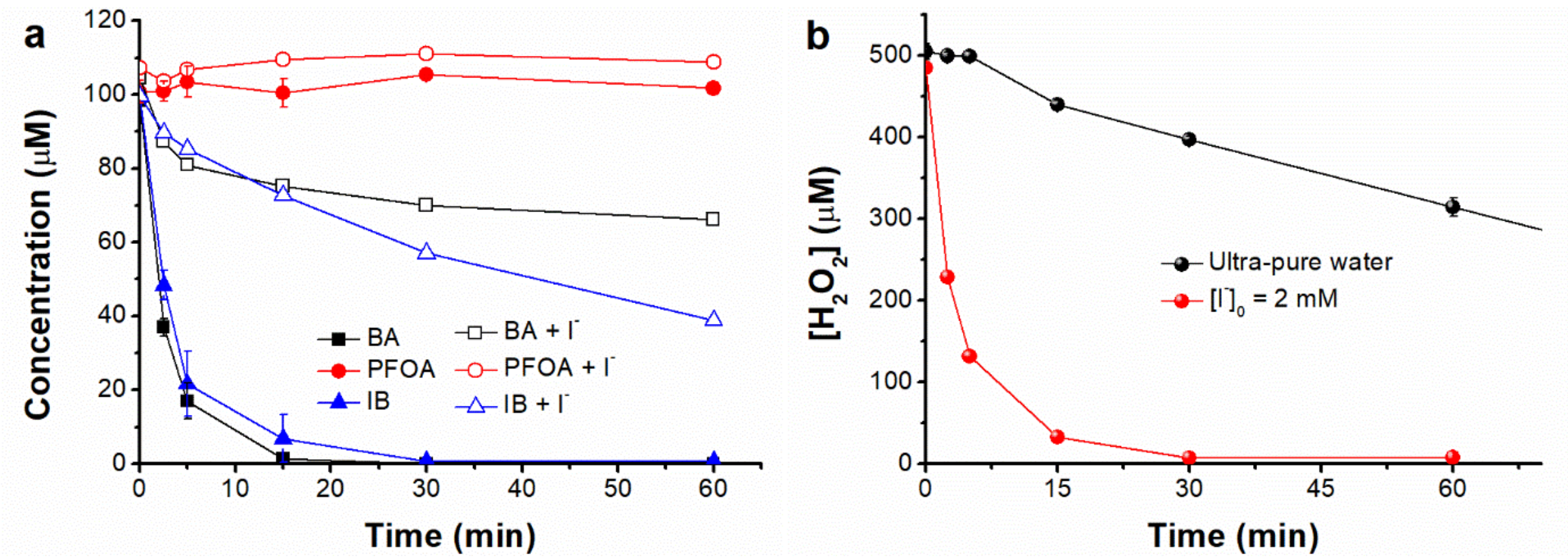

Figure S12. Effect of I^- 2 mM on the UVC/H_2O_2 performance ($[H_2O_2]_0 = 0.5$ mM, $pH_0 = 7.0$): a) pollutants and b) H_2O_2 concentration kinetics, respectively.

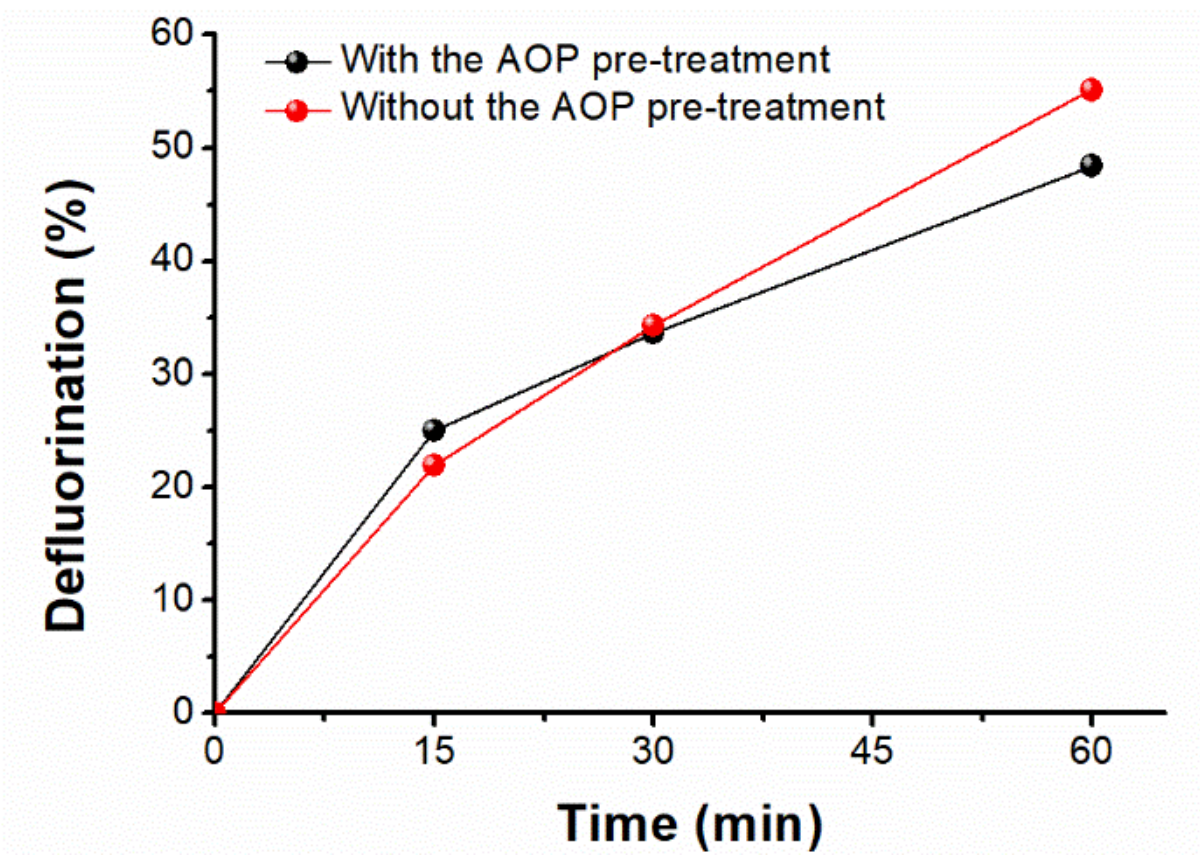

Figure S13. AOP→ARP treatment train in ultra-pure water. The AOP pre-treatment consisted of UVC/H_2O_2 process ($[H_2O_2]_0 = 5$ mM, pH 7.0) for 2 h, and the ARP post-treatment the UVC/SO_3^{2-}/I^- ($[SO_3^{2-}]_0 = 10$ mM, $[I^-]_0 = 2$ mM, $pH_0 = 12.0$, N_2 atmosphere).

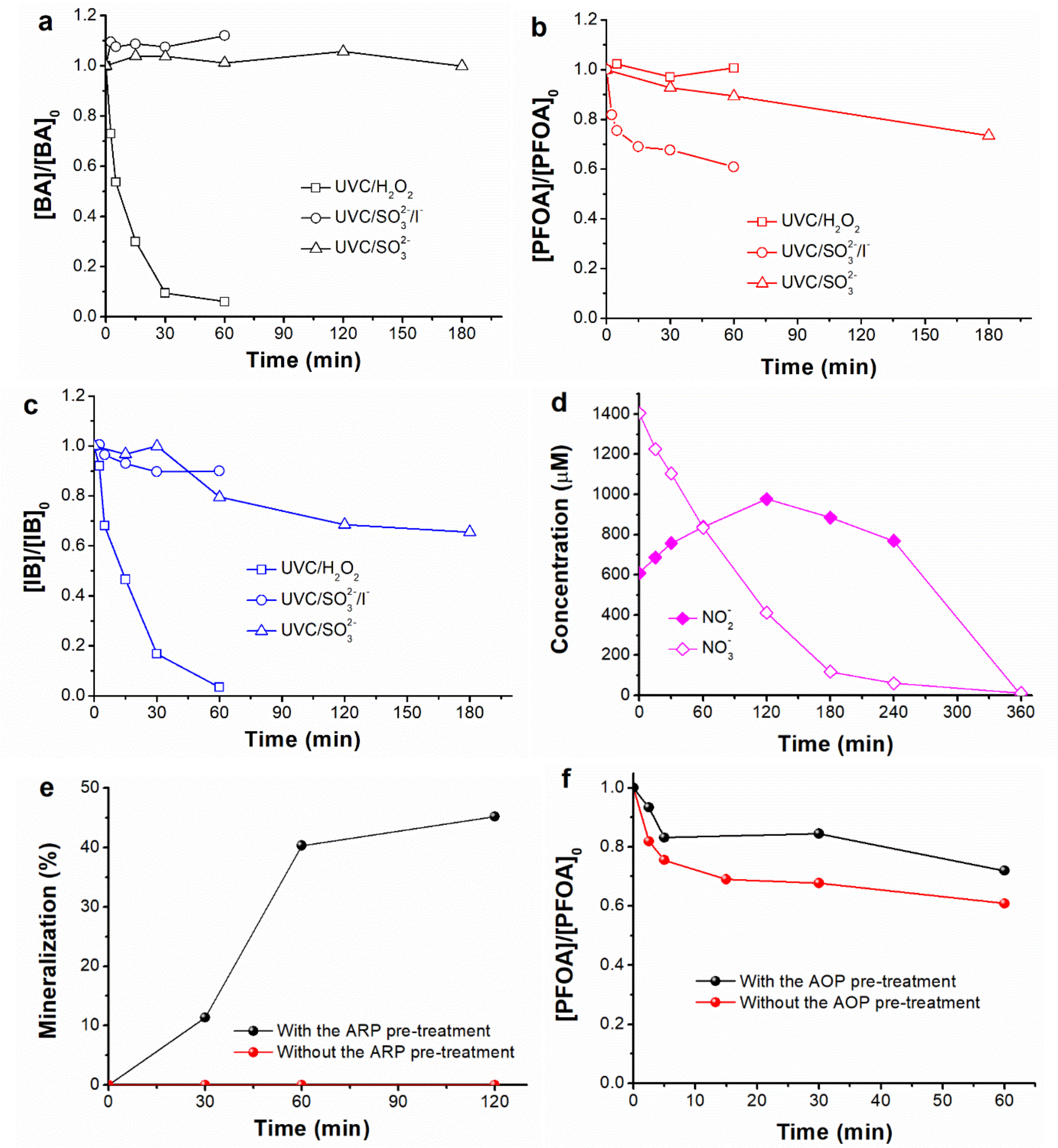

Figure S14. Results in simulated retentate wastewater: a), b) and c) BA, IB and PFOA concentration trends obtained with the different studied processes, d) NO_2^- and NO_3^- concentration changes over time during the UVC/SO_3^{2-} process, e) mineralization results by UVC/H_2O_2 applied with and without the UVC/SO_3^{2-} pre-treatment of 6 h, f) PFOA removal by UVC/SO_3^{2-}/I^- alone and after UVC/H_2O_2 pre-treatment of 2 h. Conditions: AOP, $[H_2O_2]_0$ = 5 mM, non-adjusted pH; ARPs, $[SO_3^{2-}]_0$ = 10 mM, $[I^-]_0$ = 2 mM, pH_0 = 12.0, N_2 atmosphere. In all cases, the initial solution contained 1 μM of BA, IB and PFOA each.

Text S1. Calculation of steady state concentrations of e_{aq}^- and $HO^\bullet$ during the degradation of BA, IB and PFOA 100 µM by the studied processes, and their changes with the presence of NO_2^- 1 mM.

For the ARPs, the e_{aq}^- consumption can be written as follows:

$$\frac{d[e_{aq}^-]}{dt} = R - \sum k_i[e_{aq}^-][X_i]$$

Being *R* the e_{aq}^- generation rate by UVC/SO_3^{2-}/I^- process, and X_i an e_{aq}^- scavenger. Assuming steady state approximation for e_{aq}^-:

$$\frac{d[e_{aq}^-]}{dt} = 0 \leftrightarrow R = [e_{aq}^-]_{ss}\sum k_i[X_i]$$

Therefore, since R depends on the lamp's photon flux (which is constant) and, in the absence of DOM, the absorbance of the solution at 254 nm is the same, in the absence and presence of NO_2^- one can write:

$$[e_{aq}^-]_{ss}\sum k_i[X_i] = [e_{aq}^-]_{ss}'\left(\sum k_i[X_i] + k[NO_2^-]\right)$$

Being *$[e_{aq}^-]'$* the steady state concentration in the presence of NO_2^- and *$[e_{aq}^-]$* in its absence, thus:

$$\frac{[e_{aq}^-]_{ss}}{[e_{aq}^-]_{ss}'} = \frac{\sum k_i[X_i] + k[NO_2^-]}{\sum k_i[X_i]} = 1 + \frac{k[NO_2^-]}{\sum k_i[X_i]} = 1 + \frac{k[NO_2^-]}{k'[BA] + k''[PFOA] + k'''[IB]}$$

Replacing with the reported bimolecular rate constants,[5–7] $k = 4 \times 10^9\ M^{-1}\ s^{-1}$, $k' = 3 \times 10^9\ M^{-1}\ s^{-1}$, $k'' = 7 \times 10^8\ M^{-1}\ s^{-1}$, and $k''' \leq 2 \times 10^7\ M^{-1}\ s^{-1}$, the initial concentrations being, $[NO_2^-]$ = 1 mM and [BA] = [PFOA] = [IB] = 0.1 mM:

$$\frac{[e_{aq}^-]_{ss}}{[e_{aq}^-]_{ss}'} = 11.75$$

With the same rationale but for the $HO^\bullet$, neglecting the PFOA contribution since k''[PFOA] << k'[BA]+k'''[IB], and being $k = 1 \times 10^{10}\ M^{-1}\ s^{-1}$, $k' = 6 \times 10^9\ M^{-1}\ s^{-1}$ and $k''' = 1 \times 10^{10}\ M^{-1}\ s^{-1}$:

$$\frac{[HO^\bullet]_{ss}}{[HO^\bullet]_{ss}'} = 1 + \frac{0.001\,M \times 1 \times 10^{10}\,M^{-1}s^{-1}}{0.0001\,M\,(6 \times 10^9\,M^{-1}s^{-1} + 1 \times 10^{10}\,M^{-1}s^{-1})} = 7.25$$

References

(1) Park, H.; Kim, T.; Kim, J.; Kim, M.-K.; Eom, S.; Choi, Y.; Zoh, K.-D. Reductive Degradation Mechanism of Perfluorooctanoic Acid (PFOA) during Vacuum Ultraviolet (VUV) Reactions Combining with Sulfite and Iodide. *Chemosphere* **2024**, *348* (November 2023), 140759. https://doi.org/10.1016/j.chemosphere.2023.140759.

(2) Ren, Z.; Bergmann, U.; Leiviskä, T. Reductive Degradation of Perfluorooctanoic Acid in Complex Water Matrices by Using the UV/Sulfite Process. *Water Res.* **2021**, *205* (August). https://doi.org/10.1016/j.watres.2021.117676.

(3) Deng, Y.; Liang, Z.; Lu, X.; Chen, D.; Li, Z.; Wang, F. The Degradation Mechanisms of Perfluorooctanoic Acid (PFOA) and Perfluorooctane Sulfonic Acid (PFOS) by Different Chemical Methods: A Critical Review. *Chemosphere* **2021**, *283* (April), 131168. https://doi.org/10.1016/j.chemosphere.2021.131168.

(4) Song, Z.; Tang, H.; Wang, N.; Zhu, L. Reductive Defluorination of Perfluorooctanoic Acid by Hydrated Electrons in a Sulfite-Mediated UV Photochemical System. *J. Hazard. Mater.* **2013**,

262, 332–338. https://doi.org/10.1016/j.jhazmat.2013.08.059.

(5) Tian, K.; Askari, N.; Cabooter, D.; Dewil, R. Leveraging an Optimized Dosing Strategy for Enhanced Degradation of Perfluorooctanoic Acid by UV/Sulfite/Iodide: Efficacy and Mechanism. *J. Environ. Chem. Eng.* **2025**, *13* (6), 119668. https://doi.org/10.1016/j.jece.2025.119668.

(6) Buxton, G. V.; Greenstock, C. L.; Helman, W. P.; Ross, A. B. Critical Review of Rate Constants for Reactions of Hydrated Electrons, Hydrogen Atoms and Hydroxyl Radicals (•OH/•O⁻ in Aqueous Solution. *J. Phys. Chem. Ref. Data* **1988**, *17* (2), 513–886. https://doi.org/10.1063/1.555805.

(7) Maza, W. A.; Breslin, V. M.; Owrutsky, J. C. Early Steps in the Advanced Reduction Process of the Hydrated Electron: Lessons Learned from Transient Spectroscopy. *Curr. Opin. Chem. Eng.* **2024**, *44*, 101015. https://doi.org/10.1016/j.coche.2024.101015.
